# Supplementary material for: Emergent constraints on the hydrological impacts of land use and land cover change
Source: Nat Commun. 2026 Feb 18;17:2908. doi: 10.1038/s41467-026-69883-2 (PMC13031313; doi:10.1038/s41467-026-69883-2)
Supplement: Supplementary file 1 — Supplementary Information [file 41467_2026_69883_MOESM1_ESM.pdf]

Supplementary Information for

Emergent constraints on the hydrological impacts of land use and land cover change

Zefeng Chen[1*], Alessandro Cescatti[2], Ruofei Xing[3,4], Giovanni Forzieri[1]

1. *Department of Civil and Environmental Engineering, University of Florence, Florence, Italy*
2. *European Commission, Joint Research Centre, Ispra, Italy*
3. *National Key Laboratory of Water Disaster Prevention, Hohai University, Nanjing, China*
4. *Business School, Hohai University, Nanjing, China*

*Corresponding author: Dr. Zefeng Chen (zefeng.chen@unifi.it)

Contents of this file

Supplementary Texts 1 to 4

Supplementary Figures 1 to 18

Supplementary Tables 1 to 8

Supplementary References

Supplementary Text

Text 1. Test on the robustness of emergent constraint approach

To verify the robustness of our emergent constraint results, several additional experiments are performed. First, we explore whether our results are highly dependent on the Earth system models (ESMs) included. To this end, we conduct twelve sets of additional experiments, each of which leaves one specific ESM out of the emergent relationship. Results show the high inter-model correlation (all $p<0.01$) between effect of historical land use and land cover change on terrestrial evapotranspiration (δET^{LULCC}) and natural logarithm of transpiration-specific Bowen ratio ($\ln(B_{ts})$) consistently across all additional experiments (Supplementary Table 4). Meanwhile, the constrained values of δET^{LULCC} derived in all experiments distribute in a narrow range, from -0.043±0.082 mm yr^{-2} to -0.014±0.073 mm yr^{-2}, and is generally close to that obtained from the full ESM ensemble (-0.031±0.081 mm yr^{-2}) (Fig. 2b). Such high consistency between results from these additional experiments performed on reduced ensemble members and those derived based on the full ESM ensemble, strongly demonstrates that our emergent constraints are robust and not conditioned by any specific ESM.

Second, we investigate the sensitivity of the constrained estimate of δET^{LULCC} to the selection of observation-based products. We derive a twenty-eight-member ensemble of global $\ln(B_{ts})$ observation by randomly and repeatedly selecting a six-member subset from the total eight-member ensemble of observation-based estimates (Supplementary Table 5). The twenty-eight sets of constrained estimates of δET^{LULCC} are largely close to that obtained from the full ensemble of observation-based estimates (-0.031±0.081 mm yr^{-2}, Fig. 2b), further demonstrating the substantial independence of our results on the selection of observations. Meanwhile, reduction in uncertainty (expressed as relative reduction in standard deviation, RR_{σ}) in these twenty-eight sets of additional experiments ranges from 42.3% to 58.1%, which further suggests the general efficacy of emergent constraint in narrowing inter-model spread.

In addition to global-scale gridded products, we also use field observational datasets of water and energy fluxes across globally distributed 132 FLUXNET sites as

reference to constrain the modelled δET^{LULCC}. The fact that weak variations over time of ESM-modelled ln(B_{ts}) values for all site locations (Supplementary Fig. 18), gives us confidence to include available sites with different observational periods into our analysis. In addition to sensible heat (H), latent heat (LE) and evapotranspiration (ET) that are available directly from FLUXNET2015 database[1], long-term transpiration (Tr) dataset for these 132 sites is provided by Nelson et al.[2], which is derived based on the combination of eddy covariance data from FLUXNET2015 with three different Tr estimation methods. These three methods for estimating Tr from eddy covariance data include the underlying water use efficiency (uWUE) method[3], the Pérez-Priego method[4], and the Transpiration Estimation Algorithm (TEA) method[5]. Locations of these FLUXNET sites are shown in Supplementary Fig. 6a, with their basic information provided in Supplementary Data 1. Based on the longitude and latitude of sites, we extract modelled B_{ts} for the corresponding grid-cells within the 0.5°×0.5° spatial resolution, and further derive the site-averaged mean annual B_{ts} and ln(B_{ts}) for each ESM. As the necessary component for subsequent hierarchical emergent constraint, we compute the mean uncertainty of site-observed ln(B_{ts}), by taking account both random uncertainty from measurement and uncertainty from the Tr estimation (Supplementary Data 2). The former, i.e., the standard deviation of H and LE measurements, has been provided in FLUXNET2015 dataset, while the latter corresponds to the standard deviation of Tr estimates by the aforementioned three methods (i.e., uWUE method, Pérez-Priego method, and TEA method). Similar to results obtained from global grid-cells (Supplementary Fig. 5a), modelled value of B_{ts} averaged across 132 sites is 19.9% lower than the observed estimate (1.295±0.287 versus 1.616±0.557). Furthermore, there exists a strong inter-model relationship between global δET^{LULCC} and natural logarithm of site averaged B_{ts} (r=0.965, p<0.01) (Supplementary Fig. 6b), consistent with the assessment performed at the global scale (Fig. 2a). After applying the emergent constraint approach, the modelled δET^{LULCC} decreases from the original 0.057±0.155 mm yr^{-2} to -0.023±0.045 mm yr^{-2} (Supplementary Fig. 6c). The resulting constrained estimate of δET^{LULCC} becomes negative, whose magnitude is largely consistent with

that one constrained by 8-member ensemble based on the combination of observation-based gridded products shown in main text (Fig. 2b).

To further enhance the confidence on the robustness of the estimated inter-model relationships between $\ln(B_{ts})$ and δET^{LULCC}, we additionally apply a random sampling technique with one-thousand replications and therefore generate one-thousand data sets by randomly extracting samples from 60% of vegetated grid-cells globally without replacement for the historical period. Each set for the historical period includes twelve δET^{LULCC} estimates and twelve associated $\ln(B_{ts})$ values, each pair of which corresponds to one CMIP6 ESM. Subsequently, we compute the inter-model correlation between δET^{LULCC} and $\ln(B_{ts})$for each data set. Results show that Pearson's correlation coefficient between δET^{LULCC} and $\ln(B_{ts})$ derived based on these one-thousand data sets reaches 0.936±0.005, with p of $8.0\times10^{-6}\pm3.3\times10^{-6}$ (Supplementary Fig. 2a,b). Such consistent and significant ($p<0.01$) inter-model correlations demonstrate good agreement in global estimates regardless of sampling, and further corroborate the robustness of our results on the emergent relationship. More importantly, the performance of the emergent constraint in terms of bias correction and reduction in uncertainty is proportionally dependent on the correlation strength. The consistent strong correlations revealed here reinforce the efficiency of our emergent constraint framework in improving model-based assessment on ET response to land use and land cover change.

Although a series of dedicated sensitivity analyses has been performed to verify the robustness of emergent constraint results (Supplementary Figs. 2 and 6, and Tables 4 and 5), the potential structural similarity between models and the resulting systematic cross-model bias still may reduce this robustness[6,7]. ESMs participating in CMIP6 represent the state-of-the-art for Earth system modelling and present substantial heterogeneity in the representation of key processes and responses to forcing, particularly for complex ET response which contains both biological and physical processes (even in its previous generation CMIP5[8]). Of the twelve ESMs assessed, only CESM2 and NorESM2-LM share some model components including land surface

model (i.e., CLM5, Supplementary Table 1). Nevertheless, compared with CESM2, modifications have been made to land surface component in NorESM2-LM[9], which can be reflected by the clear difference in estimates of historical δET^{LULCC} for these two ESMs (CESM2: 0.133 mm yr^{-2}; NorESM2-LM: 0.039 mm yr^{-2}, Fig. 1b). Because of this diversity, we stressed that shared structural similarity between ESMs would not bias our results.

Text 2. Emergent constraint results derived based on B_{ts}

In this study, $\ln(B_{ts})$, instead of B_{ts}, is used to build the emergent relationship with the modelled ET response. This is primarily because the natural logarithm regression has a better performance in fitting the inter-model relationship between δET^{LULCC} and B_{ts} over 1982-2014 compared with the linear regression, as reflected by a higher determination coefficient (r^2) for the former fitting function (Supplementary Fig. 3a). To further examine whether the relationship between δET^{LULCC} and B_{ts} is inherently non-linear across models, we perform a statistical analysis by applying a random sampling technique with one-thousand replications, and therefore generate one-thousand data sets by randomly extracting samples from 60% of vegetated grid-cells globally without replacement. Each data set includes twelve δET^{LULCC} estimates and twelve associated B_{ts} values, each pair of which corresponds to one CMIP6 ESM. We then fit linear, natural logarithmic, and quadratic regressions to the inter-model relationship between δET^{LULCC} and B_{ts} for each data set, and use r^2 and root mean square error (*RMSE*) to decide the regression model that provides the best fit in each data set. We find that mean r^2 of natural logarithmic regression fitting reaches 0.878 and is higher than that of linear and quadratic ones (0.851 and 0.876) (Supplementary Fig. 4a). In addition, mean *RMSE* of natural logarithmic regression fitting is 0.061 mm yr^{-2}, which is lower when compared to the fitting by linear and quadratic regressions (0.067 mm yr^{-2} and 0.063 mm yr^{-2}) (Supplementary Fig. 4b). As natural logarithmic regression has the best performance in fitting the inter-model relationship between δET^{LULCC} and B_{ts}, $\ln(B_{ts})$ is used in our emergent constraint framework as reference.

To further increase the confidence in our approach, we also compute and compare

the inter-model correlation between δET^{LULCC} and $\ln(B_{ts})$, against that between δET^{LULCC} and B_{ts}, during one-thousand replications of random sampling similar to that described above. The Pearson's correlation coefficient between δET^{LULCC} and $\ln(B_{ts})$ derived based on these one-thousand data sets reaches 0.936±0.005, higher than that one between δET^{LULCC} and B_{ts} (0.922±0.006) (Supplementary Fig. 2a). Given the proportional relation between correlation strength and the performance of emergent constraint, these additional analyses further confirm the utility of applying a log-transformation to B_{ts} in emergent relationship.

Moreover, to test whether applying the log-transformation or not would produce substantially different emergent constraint results, we replicate the analyses by replacing $\ln(B_{ts})$ with B_{ts}, and compare the constrained estimates derived based on B_{ts} with the original ones described in the main text (i.e., based on $\ln(B_{ts})$). Results based on B_{ts} show that the constrained δET^{LULCC} reaches -0.030±0.072 mm yr^{-2} at the global scale during the period 1982-2014, highly consistent with that one derived from $\ln(B_{ts})$ (-0.031±0.081 mm yr^{-2}) (Fig. 2 and Supplementary Fig. 14a,b). We further compare the constrained estimates derived based on B_{ts} against those derived based on $\ln(B_{ts})$ at the regional scale. Results show the general agreement between these two sets of estimates, as reflected by the fact that the absolute differences in their magnitudes are very small in all regions (Fig. 3 and Supplementary Fig. 15a). Such high consistency of results derived based on B_{ts} and those based on $\ln(B_{ts})$ indicates that the choice of the B_{ts} representation (i.e., log-transformation or not) has a negligible impact on our results and further demonstrates the robustness of our constraints on hydrological impact assessments.

Text 3. Results obtained within the classic emergent constraint framework

In addition to the hierarchical emergent constraint proposed by Bowman et al.[10], we perform additional analysis replicated by using the classic emergent constraint, which has also been widely applied in recent studies[11,12]. Associated estimates are then compared with analogous estimates obtained based on the hierarchical emergent constraint described in the main text, to further verify the robustness of our constrained

results. To this end, following the approach reported in Winkler et al.[12], we apply bootstrapping to estimate the 68% confidence of the emergent linear relationship between δET^{LULCC} and $\ln(B_{ts})$ across ESMs. Specifically speaking, we randomly resample the data with replacement, where the size of the resample is equal to the size of the original resample (i.e., twelve). We then compute the least-squares linear best fit for the resampled data, and repeat this procedure one-thousand times, and further derive the 68% confidence contours of equal probability based on the set of one-thousand random regression lines. By combining this 68% confidence contours estimated by bootstrapping with $\ln(B_{ts})$ observations and associated uncertainty, we finally derive the probability density function of the classic emergent constraint in δET^{LULCC}. Results based on the classic emergent constraint show that historical LULCC has led to a decrease in ET with a global average rate of -0.048 mm yr^{-2} during the period 1982-2014, which is generally consistent with the current estimate derived based on the hierarchical emergent constraint (-0.031 mm yr^{-2}) (Fig. 2 and Supplementary Fig. 7a,b). The high consistency emerging among the two sets of results demonstrates the substantial independence of our results on the selection of emergent constraint type. Moreover, both sets of results consistently show a reversal in the sign of original ESM estimates (0.057 mm yr^{-2}), further reinforcing the robustness of our constrained results about the negative value of global δET^{LULCC}.

It should be noted that the inter-model spread after applying the classic emergent constraint ranges between -0.136 and 0.042 mm yr^{-2} expressed by the 68% interval assuming a Gaussian distribution, and corresponds to the standard deviation of 0.089 mm yr^{-2} (Supplementary Fig. 7b). The wider spread obtained when replacing the hierarchical emergent constraint by the classic one (0.081 mm yr^{-2} vs. 0.089 mm yr^{-2}) is consistent with findings reported in previous literature[10]. Such better performance of hierarchical emergent constraint arises from its analytical Bayesian conditioning and the absence of bootstrap-induced resampling noise. To minimize the uncertainty and further derive a more robust ESM-based assessment on the hydrological effect of LULCC, we therefore adopt the hierarchical emergent constraint instead of the classic one.

Text 4. Details in constrained projections of global impact of future afforestation

Our results derived based on nine CMIP6 ESMs show a significantly negative inter-model relationship between modelled future (2015-2099) afforestation impact on terrestrial evapotranspiration (δET^{AFF}) and modelled historical (1982-2014) $\ln(B_{ts})$ at the global scale (r=-0.878, p<0.01), suggesting that the emergent constraint approach can also be used to correct the ESM-based projections of the hydrological impact of future afforestation (Supplementary Fig. 9a). Based on such relationship, we subsequently derive an observationally constrained estimate of future δET^{AFF} of 0.051±0.031 mm yr^{-2}, which is much higher than the estimate derived from the original CMIP6 ensemble mean and shows a reduction in uncertainty of 35.6% (unconstrained δET^{AFF}: 0.024±0.048 mm yr^{-2}, Supplementary Fig. 9b).

The resulting constrained estimate of δET^{AFF} described above is further used to quantify the concomitant afforestation-induced P change (δP^{AFF}) based on the significant positive correlation between these modelled δET^{AFF} and δP^{AFF} across ESMs (r=0.707, p<0.05) (Supplementary Fig. 9c). Results show that constrained global δP^{AFF} is projected to be positive during the period 2015-2099, with the mean magnitude of 0.022±0.038 mm yr^{-2} (Supplementary Fig. 9d). Such constrained value of δP^{AFF} is substantially different from the unconstrained one (-0.011±0.084 mm yr^{-2}), both in terms of sign and magnitude (Supplementary Fig. 9d). By combining the observationally constrained estimates of δET^{AFF} and δP^{AFF} (Eq. (11), details in Methods), we find that the afforestation-induced reduction in terrestrial water availability ($\delta WA^{AFF} = \delta P^{AFF} - \delta ET^{AFF}$) through enhancing ET is expected to be partly mitigated by the positive feedback on P, with an overall offset of 42.8%. The mitigation effect of the increased P on the afforestation-induced water resource depletion can therefore be attributed to the enhanced atmospheric moisture recycling following large-scale afforestation[13,14]. It is worth noting that unconstrained model outputs, despite their weaker positive δET^{AFF}, show a negative δP^{AFF} (-0.011±0.084 mm yr^{-2}) (Supplementary Fig. 9b,d), and correspondingly project a severer decline in WA due to afforestation compared with constrained results (unconstrained δWA^{AFF}: -0.035 mm yr-

^{2}; constrained δWA^{AFF}: -0.029 mm yr^{-2}).

To verify the robustness of our emergent constraint results derived under future afforestation scenario, we also conduct nine sets of additional experiments, each of which leaves one specific ESM out of the emergent relationship. Results show high inter-model correlation between $\ln(B_{ts})$ and δET^{AFF} (all p<0.01) and high correlation between δET^{AFF} and δP^{AFF} (p<0.05 for seven out of nine, and p<0.10 for the remaining two) across all additional experiments (Supplementary Table 6). Furthermore, the constrained values of δET^{AFF} derived in all experiments distribute in a narrow range, from 0.047±0.032 mm yr^{-2} to 0.055±0.033 mm yr^{-2}, and it is generally close to that obtained from the full ESM ensemble (0.051±0.031 mm yr^{-2}) (Supplementary Fig. 9b). Such high consistency can also be found in the constrained projections of δP^{AFF} and δWA^{AFF} (Supplementary Table 6).

To further enhance the confidence on the robustness of the estimated inter-model relationships between $\ln(B_{ts})$ and δET^{AFF}, additional experiments are performed through random sampling over global grid-cells similar to that for the historical period (details in Supplementary Text 1). Each set for the future scenario period includes nine δET^{AFF} estimates and nine associated $\ln(B_{ts})$ values, each pair of which corresponds to one CMIP6 ESM. We then compute the inter-model correlation between δET^{AFF} and $\ln(B_{ts})$ for each data set. Results show that Pearson's correlation coefficient between δET^{AFF} and $\ln(B_{ts})$ is -0.878±0.006, with p of $18.6\times10^{-4}\pm3.0\times10^{-4}$ (Supplementary Fig. 2c,d), further demonstrating the robustness of our results about the significant inter-model relationship between $\ln(B_{ts})$ and δET^{AFF}. Moreover, random sampling technique is also applied to verify the robustness of tight inter-model correlation between δET^{AFF} and δP^{AFF}. Results show the consistent strong correlation between δET^{AFF} and δP^{AFF} over one-thousand sets (Supplementary Fig. 2e,f), highlighting the reliability of deriving more accurate assessment on future afforestation impact on WA inferring from the constrained estimate of δET^{AFF}.

Following sensitivity analyses performed for the historical period, we additionally test whether replacing $\ln(B_{ts})$ with B_{ts} would produce substantially different emergent

constraint results under future afforestation scenario. We find that similar to δET^{LULCC} results, δET^{AFF} also exhibits a stronger correlation with $\ln(B_{ts})$ across models compared to that one obtained with B_{ts} (-0.878±0.006 vs. -0.866±0.006) (Supplementary Fig. 2a,c). Global-mean values of δET^{AFF}, δP^{AFF} and δWA^{AFF} after the B_{ts}-based constraint are 0.052 mm yr^{-2}, 0.024 mm yr^{-2} and -0.028 mm yr^{-2}, respectively, which are also in accordance with those after the $\ln(B_{ts})$-based constraint (0.051 mm yr^{-2}, 0.022 mm yr^{-2} and -0.029 mm yr^{-2}) (Supplementary Figs. 9 and 14c,d). Such high consistency between two sets of estimates can also be found at the regional scale (Fig. 5a and Supplementary Fig. 15b).

Finally, we also replicate the analyses under future afforestation scenario by using the classic emergent constraint[11,12], and compare associated constrained estimates against analogous estimates derived based on the hierarchical emergent constraint shown in Fig. 2. Results based on the classic emergent constraint show the afforestation-induced ET increase of 0.060±0.034 mm yr^{-2} globally during the period 2015-2099, which is highly consistent with constrained estimate of δET^{AFF} derived from the hierarchical emergent constraint (0.051±0.031 mm yr^{-2}) (Supplementary Figs. 7c,d and 9a,b). Meanwhile, the higher value of constrained δET^{AFF} by the classic emergent constraint compared with the original ESM simulation (0.024±0.048 mm yr^{-2}) further supports our finding that current-version ESMs generally underestimate the positive effect of afforestation on ET at the global scale (Supplementary Figs. 7d).

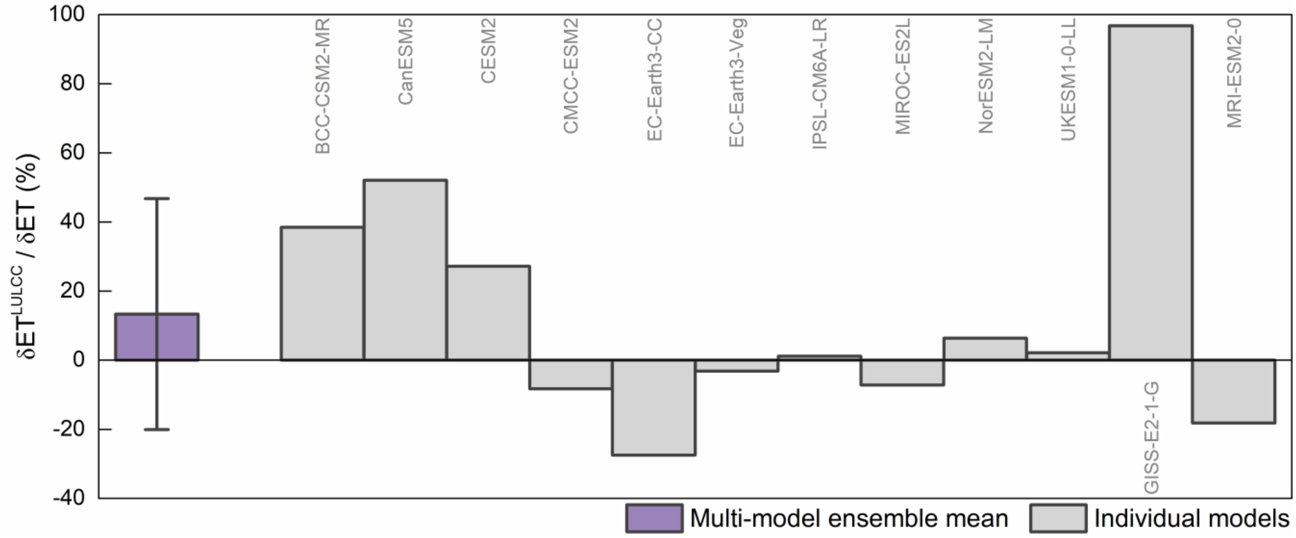

Supplementary Fig. 1. Relative contribution of land use and land cover change on trend in terrestrial evapotranspiration during the period 1982-2014. Error bars represent the standard deviation of relative contributions derived from ensemble members (i.e., twelve CMIP6 ESMs).

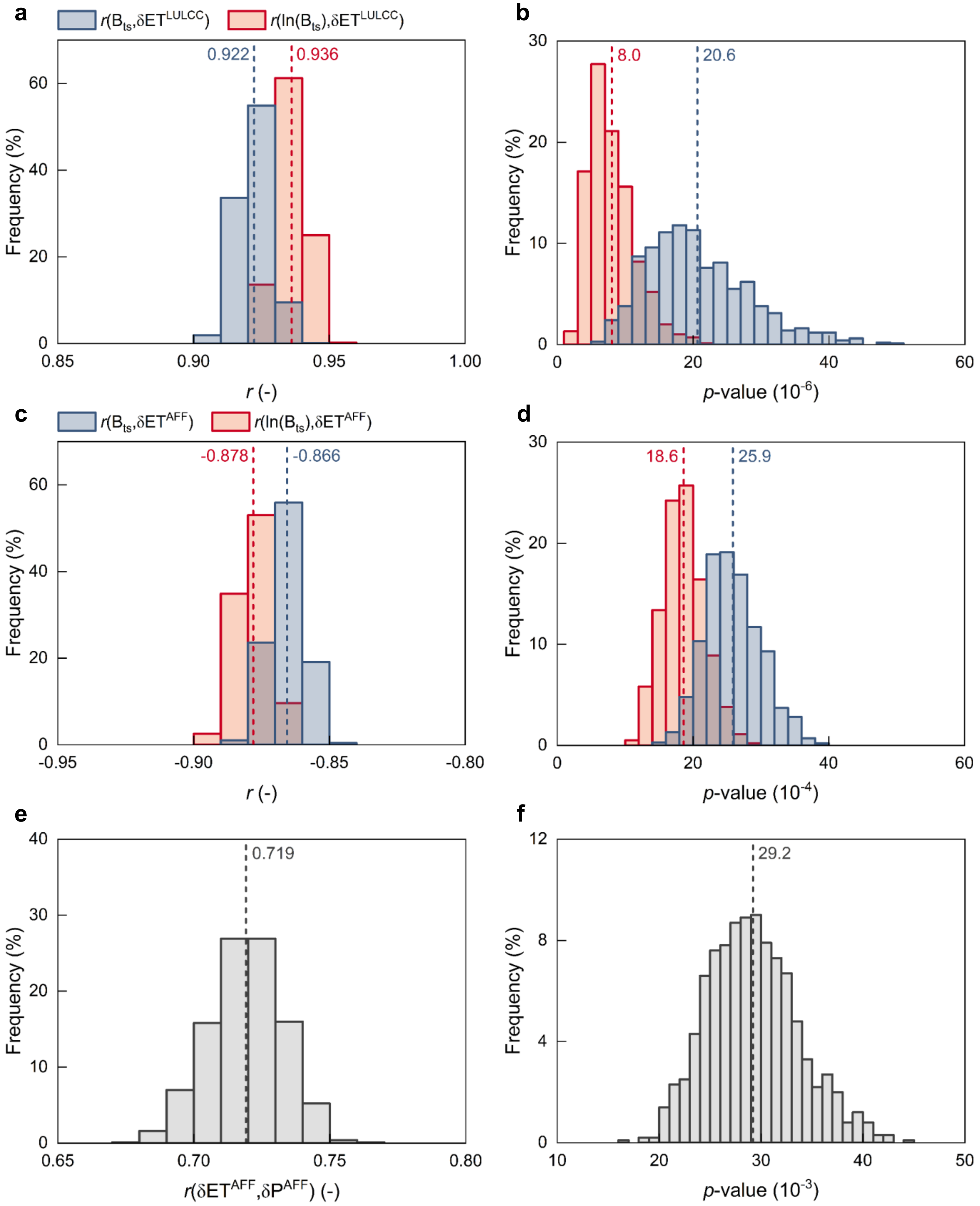

Supplementary Fig. 2. Inter-model correlation and associated significance level for one-thousand random sampling. (a) Frequency distribution of one-thousand sets of inter-model correlation (r) between the effect of historical land use and land cover change (generally characterized as forest-cropland conversion) on annual evapotranspiration (δET^{LULCC}) and the global averaged transpiration-specific Bowen ratio (B_{ts}), as well as r between δET^{LULCC} and natural logarithm value of B_{ts} (i.e., $\ln(B_{ts})$) during the period 1982-2014. Each data set is generated by randomly extracting samples from 60% of vegetated grid-cells globally without replacement. Each set includes twelve δET^{LULCC} estimates and twelve associated B_{ts} values, each pair of which corresponds to one CMIP6 ESM. Distribution averages are shown as dashed horizontal lines. (b) Same as (a),

but for the *p*-value associated to the inter-model correlation coefficient between δET^{LULCC} and B_{ts}, and that between δET^{LULCC} and $\ln(B_{ts})$. (c and d), Same as (a and b), but for *r* with the effect of future (2015-2099) afforestation on annual evapotranspiration (δET^{AFF}), and the associated *p*-value. During one-thousand random sampling procedure, each set includes nine δET^{AFF} estimates and nine associated B_{ts} values, each pair of which corresponds to one CMIP6 ESM. (e and f), Same as (a and b), but for *r* between δET^{AFF} and the modelled effect of future (2015-2099) afforestation on annual precipitation (δP^{AFF}), and the associated *p*-value.

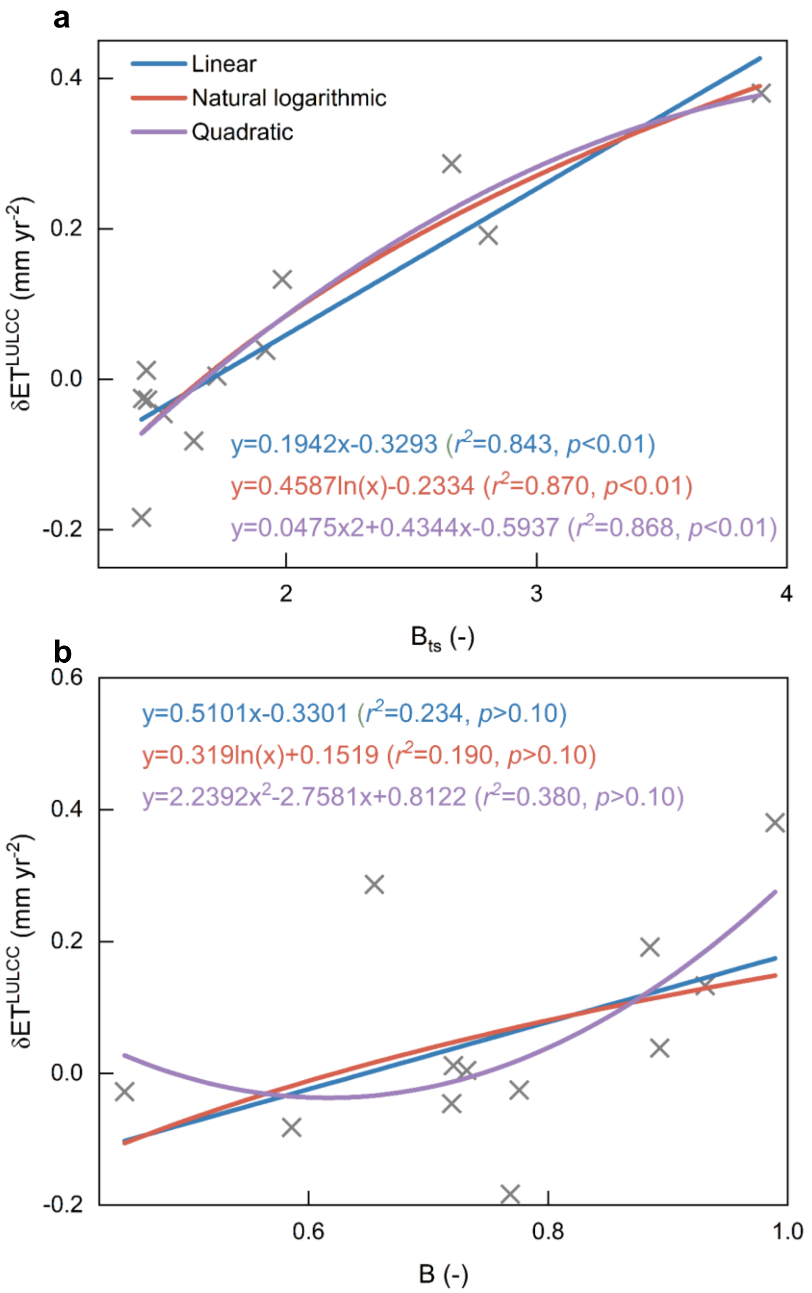

Supplementary Fig. 3. Best fit of the underlying inter-model relationship between the surface energy partitioning and the effect of historical land use and land cover change on terrestrial evapotranspiration. (a) Transpiration-specific Bowen ratio (B_{ts}) and the effect (δET^{LULCC}) during the period 1982-2014 are derived from simulations of twelve ESMs. Each symbol denotes a CMIP6 ESM result. Linear, natural logarithmic, and quadratic regression models are used to fit such relationship, respectively. Performance of three regression models is evaluated by determination coefficient (r^2). (b) Same as (a), but for standard Bowen ratio (B).

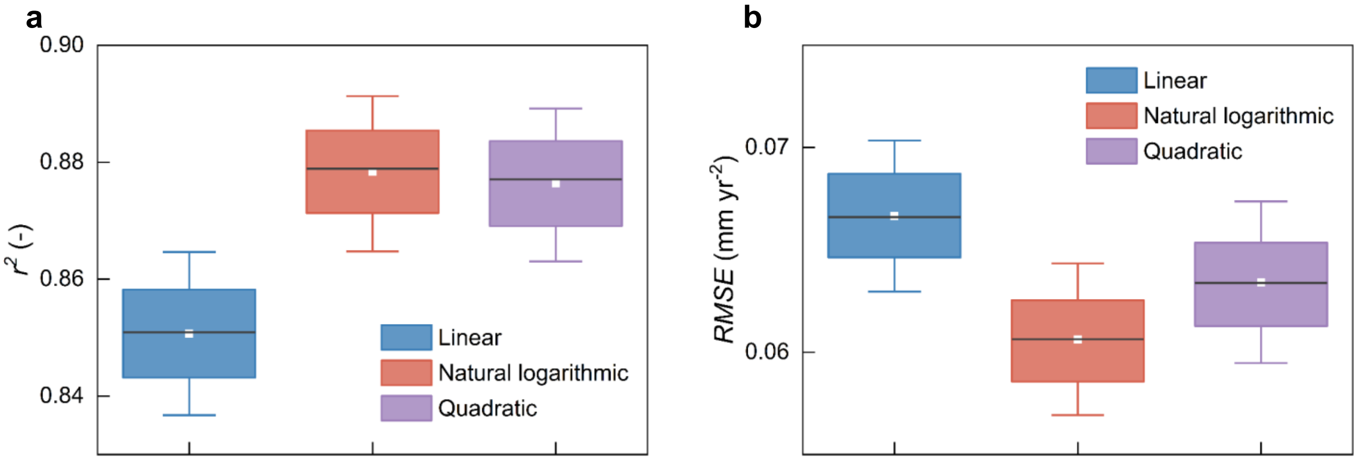

Supplementary Fig. 4. Performance comparison of different regression model types in fitting the inter-model relationship for one-thousand random sampling. (a) Boxplot of one-thousand sets of determination coefficient (r^2) of linear, natural logarithmic, and quadratic regression models in fitting the inter-model relationship between the effect of land use and land cover change on annual evapotranspiration (δET^{LULCC}) and the global averaged transpiration-specific Bowen ratio (B_{ts}) during the period 1982-2014. Each data set is generated by randomly extracting samples from 60% of vegetated grid-cells globally without replacement. Each set includes twelve δET^{LULCC} estimates and twelve associated B_{ts} values, each pair of which corresponds to one CMIP6 ESM. Boxplot elements: box = values of 25th and 75th percentiles; horizontal line = median; rectangle = mean; whiskers = values of 10th and 90th percentiles. (b) Same as (a), but for root mean square error (*RMSE*).

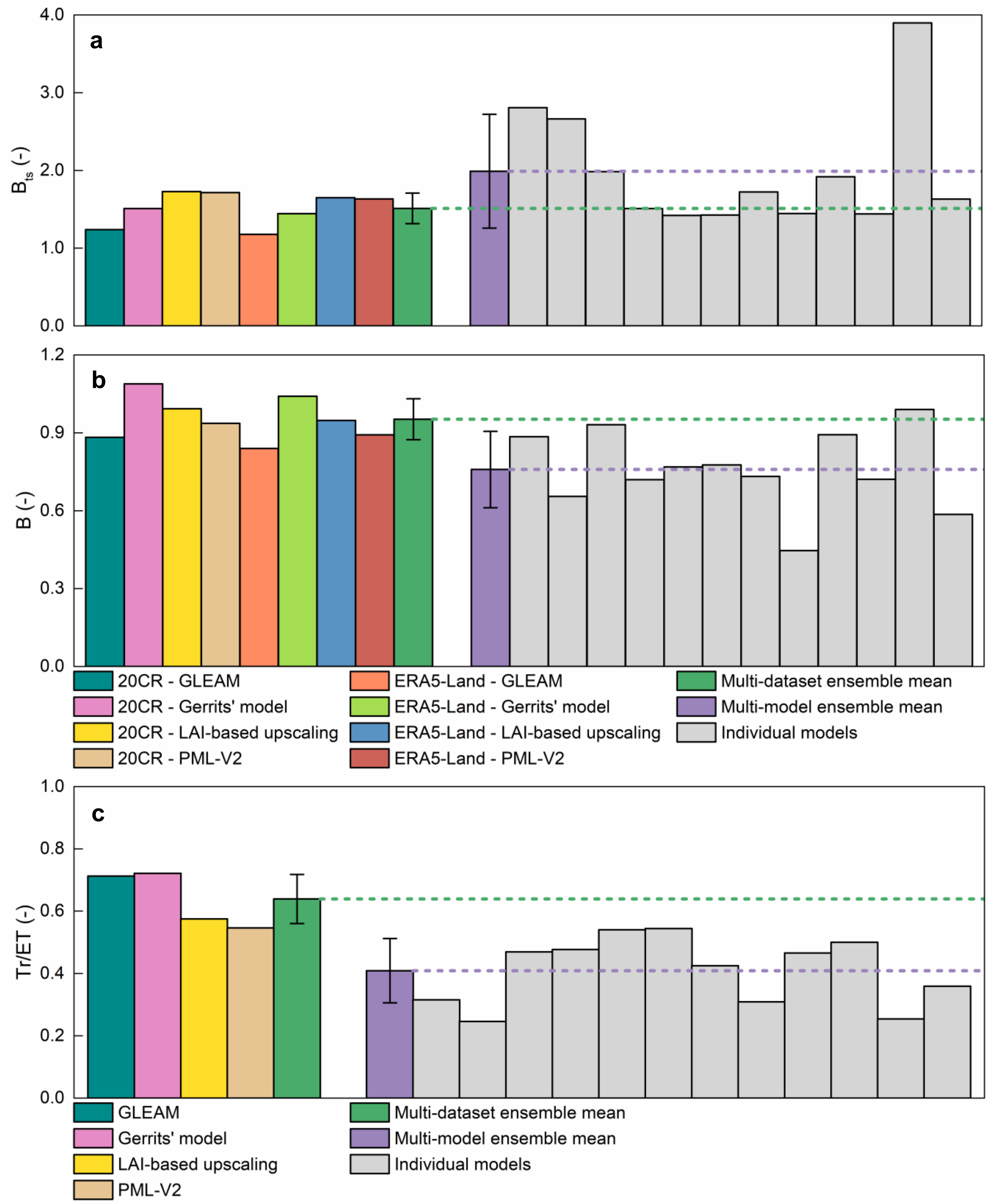

Supplementary Fig. 5. Global averaged transpiration-specific Bowen ratio (B_{ts}) and its components during the period 1982-2014, derived from multiple observation-based combined datasets and CMIP6 ESMs and their ensemble mean. (a) B_{ts}, (b) the original Bowen ratio (B), and (c) the ratio of transpiration to total terrestrial evapotranspiration (Tr/ET). B_{ts} is derived by dividing B by Tr/ET (i.e., Eq. (4)). Error bars represent the standard deviation of variables derived from ensemble members (i.e., eight combined datasets for multi-dataset ensemble mean; twelve individual ESMs for multi-model ensemble mean), respectively. For Tr/ET, multi-dataset ensemble mean and its standard deviation are estimated based on four individual datasets.

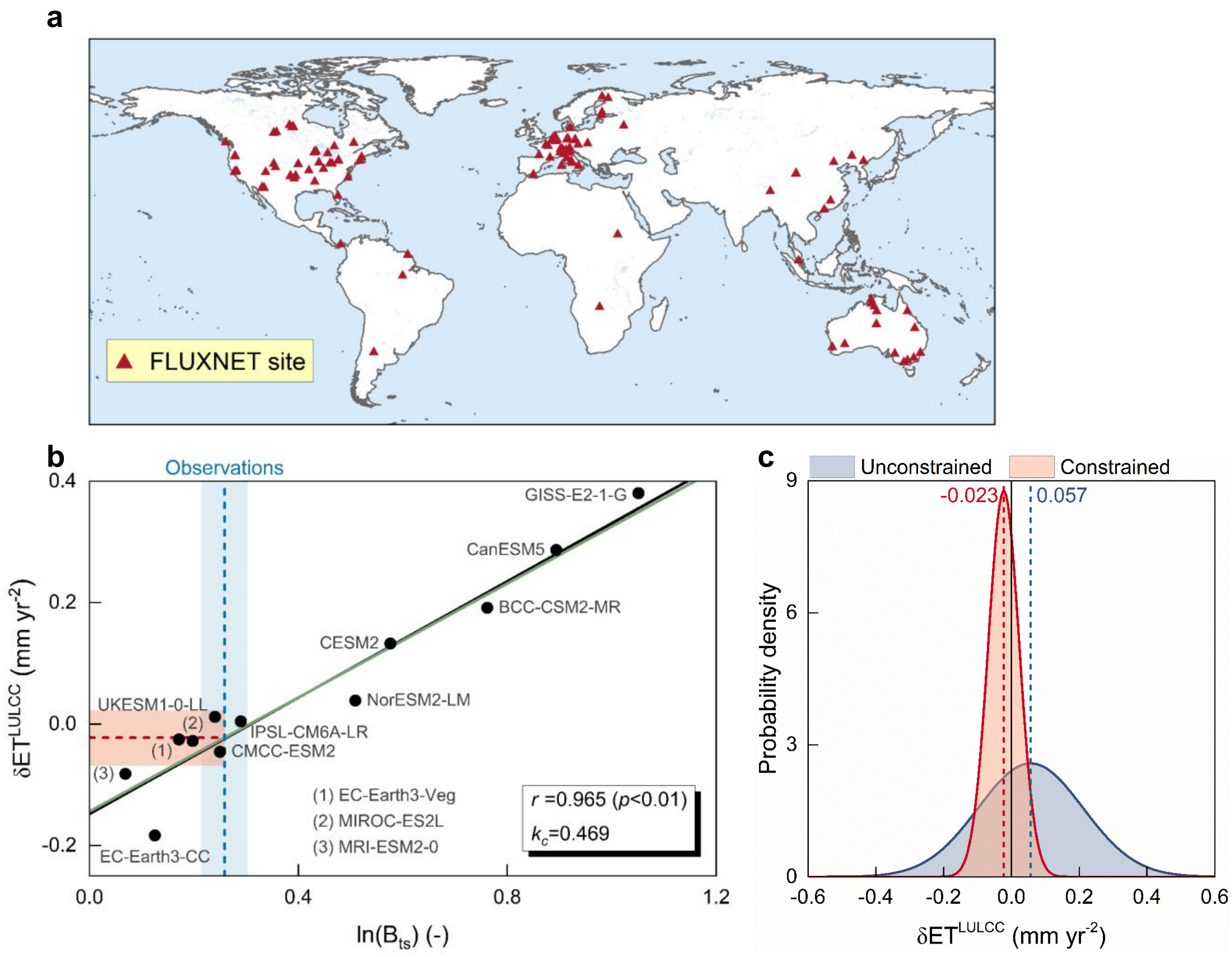

Supplementary Fig. 6. A constraint on the effect of historical land use and land cover change on terrestrial evapotranspiration based on eddy covariance measurements. (a) Location of 132 FLUXNET sites that provide stand-level observations for this study. Detailed information of these sites is provided in Supplementary Data 1 and 2. (b) Emergent relationship between the modelled global averaged effect of land use and land cover change (generally characterized as forest-cropland conversion) on annual evapotranspiration (δET^{LULCC}) and the modelled natural logarithm value of cross-site averaged transpiration-specific Bowen ratio ($\ln(B_{ts})$) during the period 1982-2014. Modelled $\ln(B_{ts})$ for each site is extracted based on the grid-cell corresponding to the longitude and latitude of site within 0.5°×0.5° spatial resolution. Each dot denotes a CMIP6 ESM result, and the black solid line indicates the best-fit regression line across ESMs, with correlation coefficient (r) provided in the panel. The green solid line indicates an observational correction based on Eq. (5) with associated slope (k_c) provided in label. The vertical blue dashed line and shaded areas represent the natural logarithm of site averaged B_{ts} derived from eddy covariance data and its uncertainty (one standard deviation). The horizonal red dashed line and shaded areas show the resulting constrained estimate of δET^{LULCC} and its uncertainty based on the hierarchical emergent constraint approach (Methods). (c) The probability density functions of global averaged δET^{LULCC} for the original results of CMIP6 ESMs (blue) and the observationally constrained results (red). ArcGIS Pro was used as technical tool for data processing and visualizing this figure. However, no ESRI basemaps or proprietary ESRI datasets, and no screenshots of the ArcGIS software interface were used.

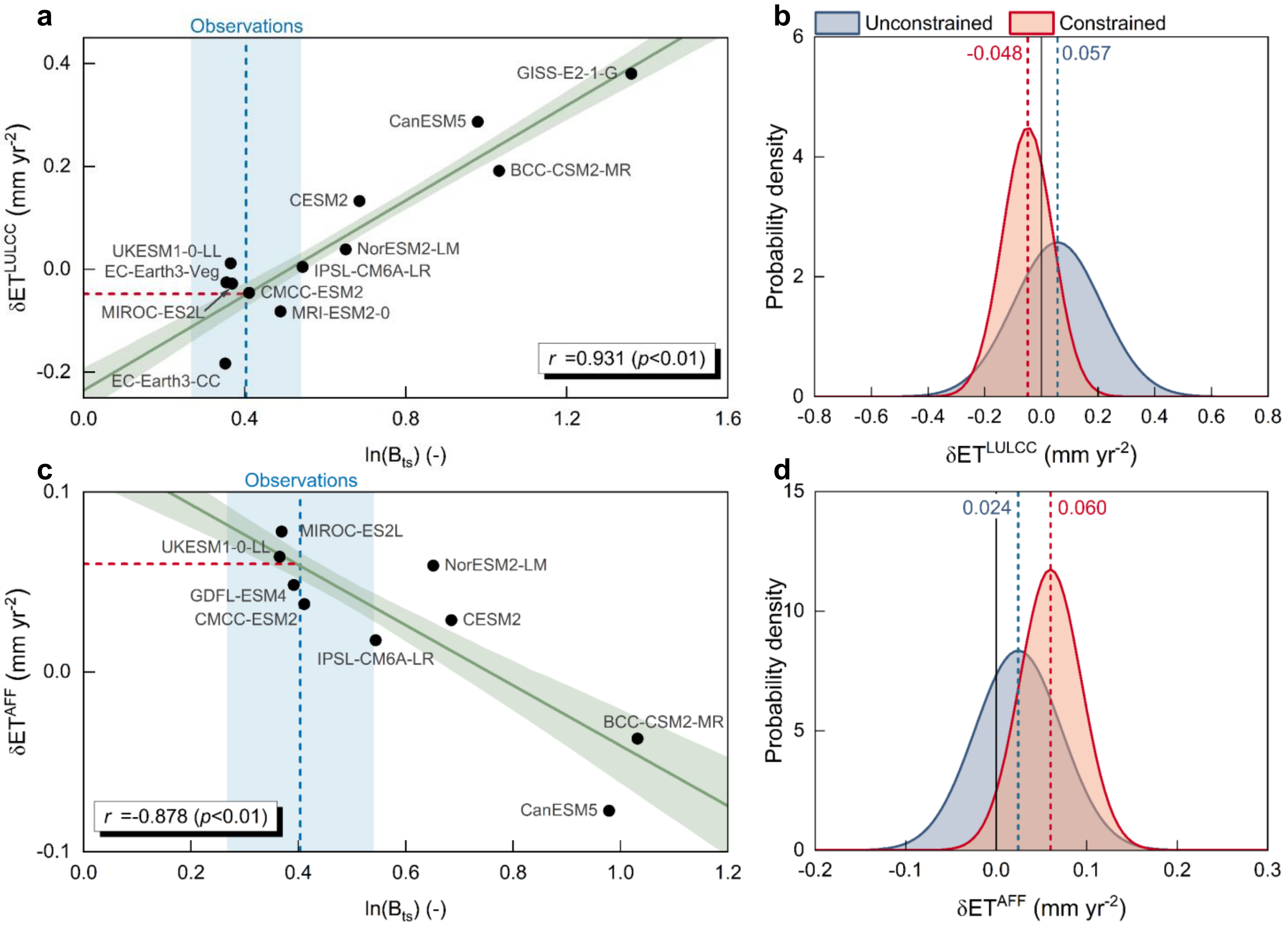

Supplementary Fig. 7. Constrained results derived based on the classic emergent constraint approach. (a) Emergent relationship between the modelled effect of land use and land cover change on annual evapotranspiration (δET^{LULCC}) and the modelled natural logarithm value of global averaged transpiration-specific Bowen ratio ($\ln(B_{ts})$) during the period 1982-2014. LULCC over this period is generally characterized as the conversion from forests to croplands (Fig. 1a). Each dot denotes a CMIP6 ESM result, which corresponds to δET^{LULCC} estimate shown in Fig. 1b. The green solid line indicates the best-fit regression line across ESMs, with correlation coefficient (r) provided in the panel. Shaded areas around the best linear fit show the 68% confidence interval estimated by bootstrapping (Supplementary Text 3). The vertical blue dashed line and shaded areas represent the observation-based estimate of $\ln(B_{ts})$ and its uncertainty (one standard deviation). Such observation-based estimate is derived from an eight-member ensemble of observation-based combined datasets (Methods). The horizonal red dashed line show the resulting constrained estimate of δET^{LULCC} based on the classic emergent constraint approach[11,12]. (b) The probability density functions of global averaged δET^{LULCC} for the original results of CMIP6 ESMs (blue) and the observationally constrained results (red). (c) and (d) Same as (a) and (b), but for the effect of future (2015-2099) afforestation on annual evapotranspiration (δET^{AFF}).

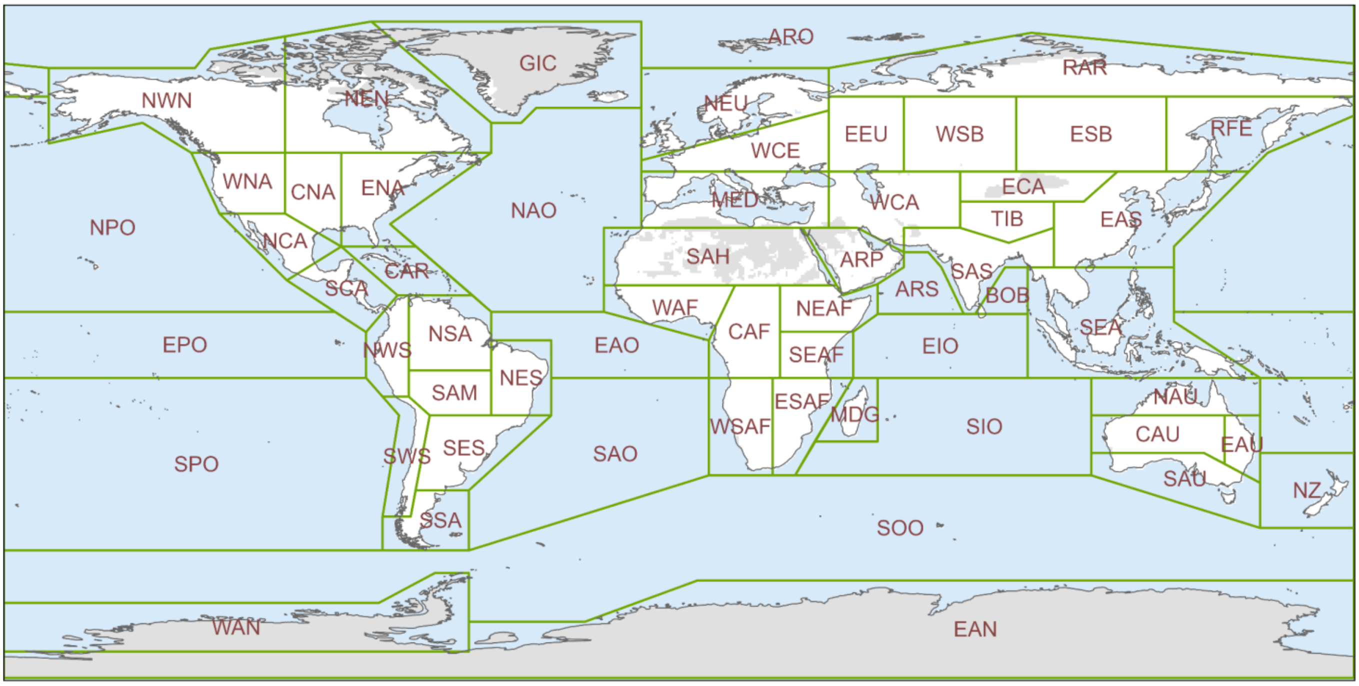

Acronym	Full name	Acronym	Full name	Acronym	Full name
GIC	Greenland/Iceland	SAH	Sahara	CAU	Central Australia
NWN	Northwestern North America	WAF	Western Africa	EAU	Eastern Australia
NEN	Northeastern North America	CAF	Central Africa	SAU	Southern Australia
WNA	Western North America	NEAF	North Eastern Africa	NZ	New Zealand
CNA	Central North America	SEAF	South Eastern Africa	EAN	Eastern Antarctica
ENA	Eastern North America	WSAF	West Southern Africa	WAN	Western Antarctica
NCA	Northern Central America	ESAF	East Southern Africa	ARO	Arctic Ocean
SCA	Southern Central America	MDG	Madagascar	NPO	North Pacific Ocean
CAR	Caribbean	RAR	Russian Arctic	EPO	Equatorial Pacific Ocean
NWS	Northwestern South America	WSB	West Siberia	SPO	South Pacific Ocean
NSA	Northern South America	ESB	East Siberia	NAO	North Atlantic Ocean
NES	Northeastern South America	RFE	Russian Far East	EAO	Equatorial Atlantic Ocean
SAM	South American Monsoon	WCA	West Central Asia	SAO	South Atlantic Ocean
SWS	Southwestern South America	ECA	East Central Asia	ARS	Arabian Sea
SES	Southeastern South America	TIB	Tibetan Plateau	BOB	Bay of Bengal
SSA	Southern South America	EAS	East Asia	EIO	Equatorial Indian Ocean
NEU	Northern Europe	ARP	Arabian Peninsula	SIO	South Indian Ocean
WCE	Western and Central Europe	SAS	South Asia	SOO	Southern Ocean
EEU	Eastern Europe	SEA	Southeast Asia		
MED	Mediterranean	NAU	Northern Australia		

Supplementary Fig. 8. IPCC AR6 WGI reference regions. Full names corresponding to acronyms shown in figure are illustrated in the following table. Non-vegetated areas, defined as multi-year (1982-2014) average leaf area index (LAI) < 0.15 m^2 m^{-2}, are excluded in our analysis and are shown in grey. It should be noted that due to the limited number (nearly zero) of valid grid-cells, two polar regions (i.e., EAN and WAN) and twelve ocean regions (i.e., ARO, NPO, EPO, SPO, NAO, EAO, SAO, ARS, BOB, EIO, SIO, and SOO) are not considered in our regional-scale analysis. Original figure and associated files are provided in Iturbide et al.[15]. ArcGIS Pro was used as technical tool for data processing and visualizing this figure. However, no ESRI basemaps or proprietary ESRI datasets, and no screenshots of the ArcGIS software interface were used.

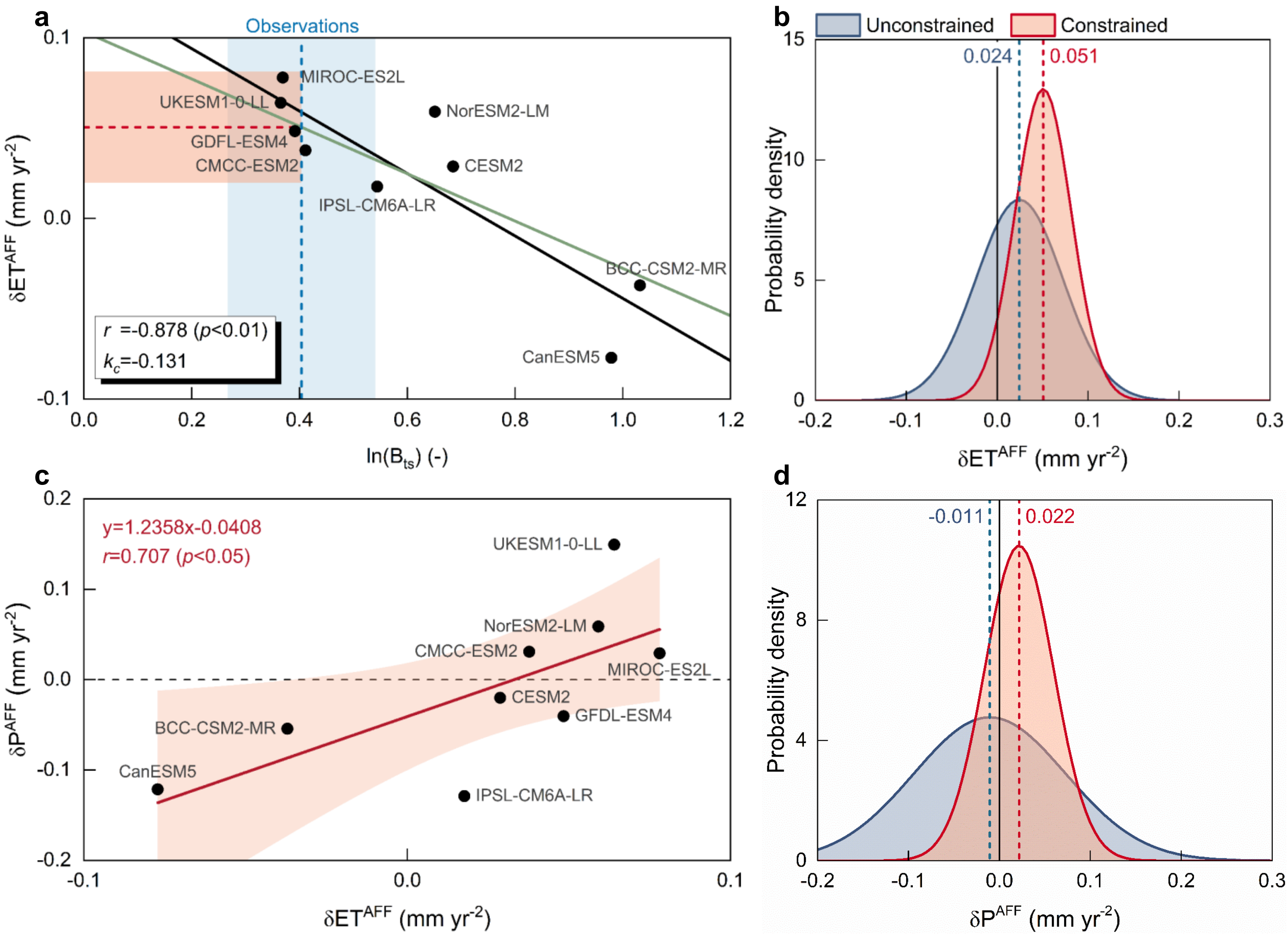

Supplementary Fig. 9. Constrained projection on global hydrological impact of future afforestation. (a) Emergent relationship between the modelled effect of future afforestation on annual evapotranspiration (δET^{AFF}) and the modelled historical (1982-2014) natural logarithm value of global averaged transpiration-specific Bowen ratio ($\ln(B_{ts})$). Each dot denotes a CMIP6 ESM result, and the black solid line indicates the best-fit regression line across ESMs, with correlation coefficient (r) provided in the panel. The green solid line indicates the observational correction based on Eq. (5) with associated slope (k_c) provided in label. The vertical blue dashed line and shaded areas represent the observation-based estimate of $\ln(B_{ts})$ and its uncertainty (one standard deviation) (Methods). The horizonal red dashed line and shaded areas show the resulting constrained estimate of δET^{AFF} and its uncertainty based on the hierarchical emergent constraint approach (Methods). (b) The probability density functions of global averaged δET^{AFF} for the original results of CMIP6 ESMs (blue) and the observationally constrained results (red). (c) Inter-model relationship between δET^{AFF} and the modelled effect on annual precipitation (δP^{AFF}). The red solid line and shaded areas indicates the best-fit regression line across ESMs and its 95% confidence range. (d) The probability density functions of δP^{AFF} for the original results of CMIP6 ESMs (blue) and the observationally constrained results (red) by Eq. (10).

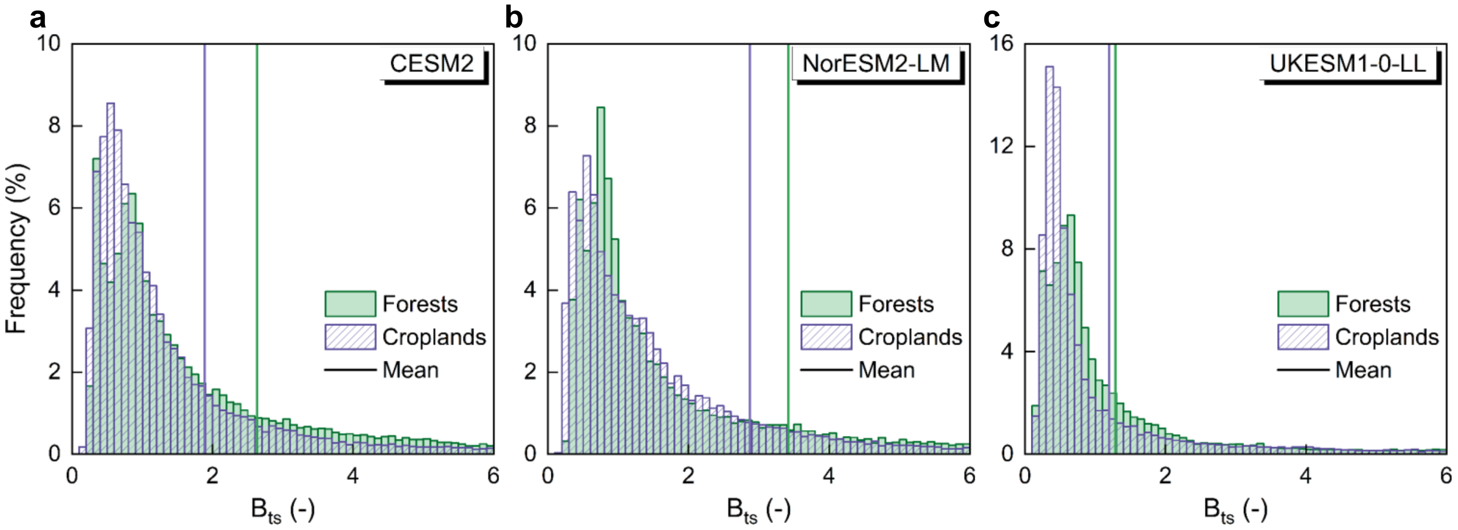

Supplementary Fig. 10. Frequency distributions of transpiration-specific Bowen ratio (B_{ts}) in forests and croplands as estimated by subgrid-scale outputs from ESMs. Three panels represent results based on outputs from (a) CESM2, (b) NorESM2-LM, and (c) UKESM1-0-LL, respectively. Distribution averages are shown as solid horizontal lines.

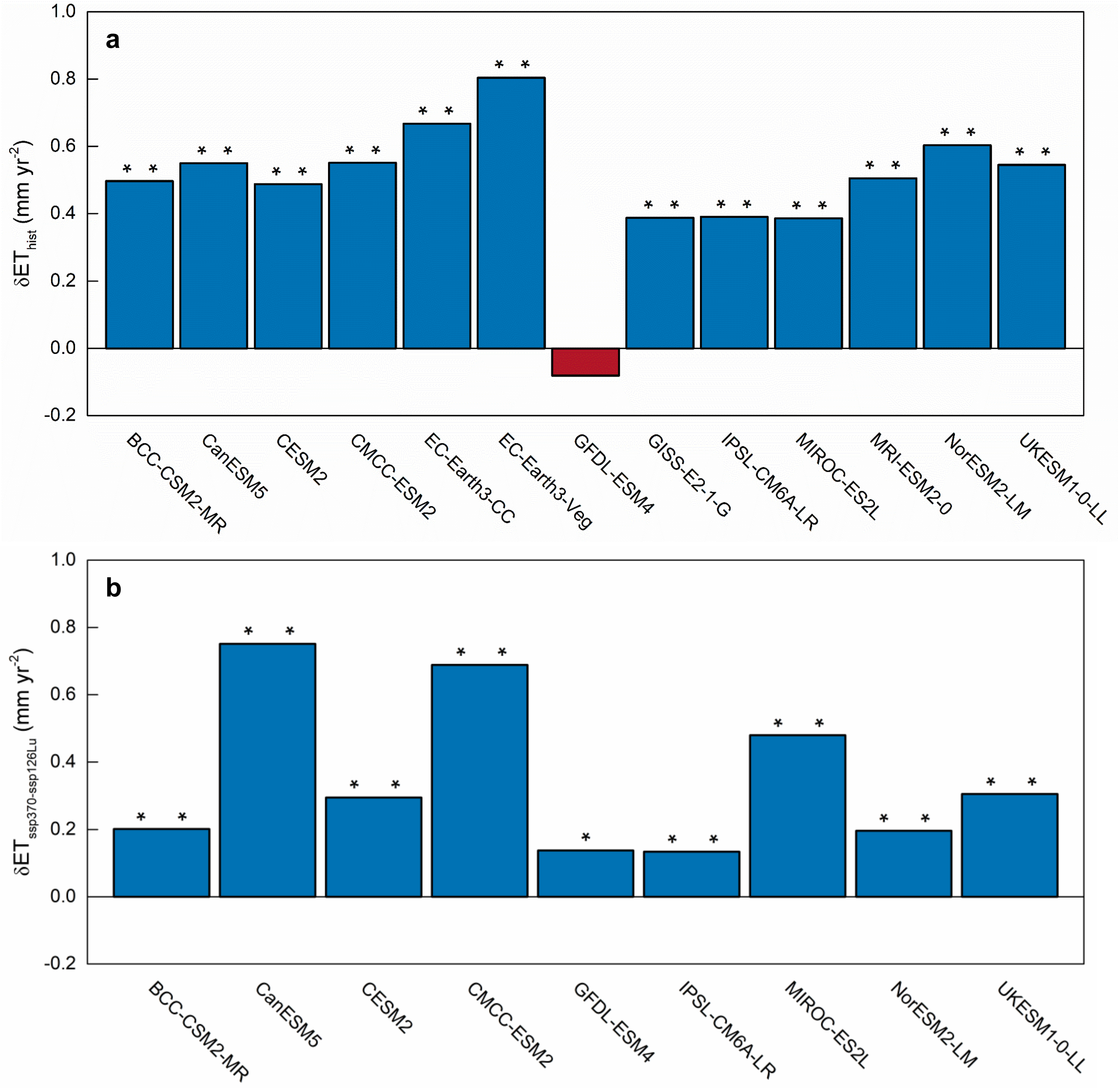

Supplementary Fig. 11. Trends in terrestrial evapotranspiration simulated by different ESMs. (a) Global mean trends in evapotranspiration (ET) during the period 1982-2014, as simulated by thirteen different ESMs in the "historical" experiment. One asterisk (*) Two asterisks (* *) indicate that the trend is statistically significant with $p<0.05$ and $p<0.01$, respectively. Significance of the trend is assessed by Mann-Kendall test. (b) Same as (a), but for global mean trends in ET during the period 2015-2099 in the "ssp370-ssp126Lu" experiment.

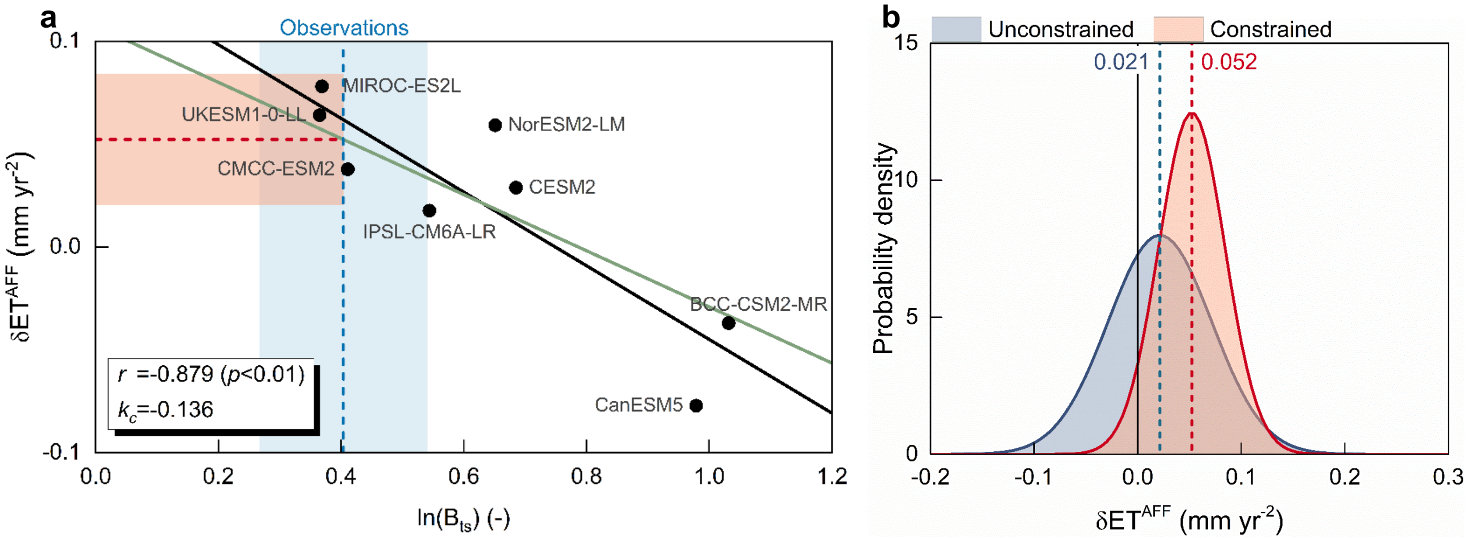

Supplementary Fig. 12. Constrained projection of future afforestation impacts on terrestrial evapotranspiration after excluding GFDL-ESM4. (a) Emergent relationship between the modelled effect of future (2015-2099) afforestation on annual evapotranspiration (δET^{AFF}) and the modelled historical (1982-2014) natural logarithm value of global averaged transpiration-specific Bowen ratio ($\ln(B_{ts})$). Each dot denotes a CMIP6 ESM result, and the black solid line indicates the best-fit regression line across ESMs, with correlation coefficient (r) provided in the panel. The green solid line indicates an observational correction based on Eq. (5) with associated slope (k_c) provided in label. The vertical blue dashed line and shaded areas represent the observation-based estimate of $\ln(B_{ts})$ and its uncertainty (one standard deviation). Such observation-based estimate is derived from an 8-member ensemble of observation-based combined datasets (Methods). The horizonal red dashed line and shaded areas show the resulting constrained estimate of δET^{AFF} and its uncertainty based on the hierarchical emergent constraint approach (Methods). (b) The probability density functions of global averaged δET^{AFF} for the original results of CMIP6 ESMs (blue) and the observationally constrained results (red).

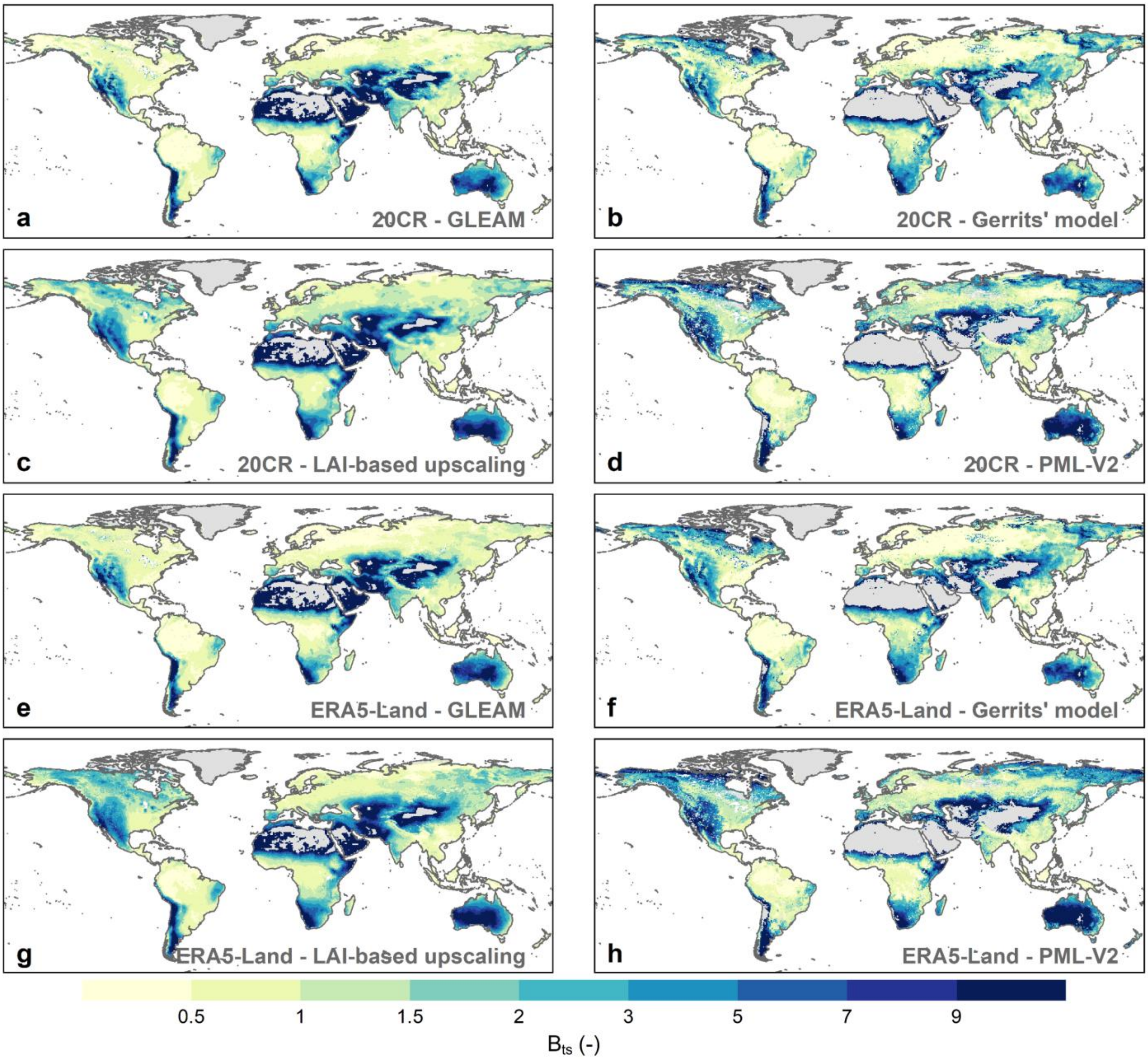

Supplementary Fig. 13. Observation-based spatial patterns of mean annual transpiration-specific Bowen ratio (B_{ts}) during the period 1982-2014. Panels show different combinations of climate reanalysis datasets and observational transpiration/evapotranspiration/ (Tr/ET) products: (a) 20CR - GLEAM; (b) 20CR - Gerrits' model; (c) 20CR – LAI-based upscaling; (d) 20CR - PML-V2; (e) ERA5-Land - GLEAM; (f) ERA5-Land - Gerrits' model; (g) ERA5-Land - LAI-based upscaling; (h) ERA5-Land - PML-V2. It is worth noting that in (b,d,f,h), B_{ts} is marked without value in extremely arid regions such as Sahel, Arabian Peninsula, and Northwestern China. This is because mean annual value of Tr in Gerrits' model and in PML-V2 equals zero in these regions, making the corresponding B_{ts} value infinite. ArcGIS Pro was used as technical tool for data processing and visualizing this figure. However, no ESRI basemaps or proprietary ESRI datasets, and no screenshots of the ArcGIS software interface were used.

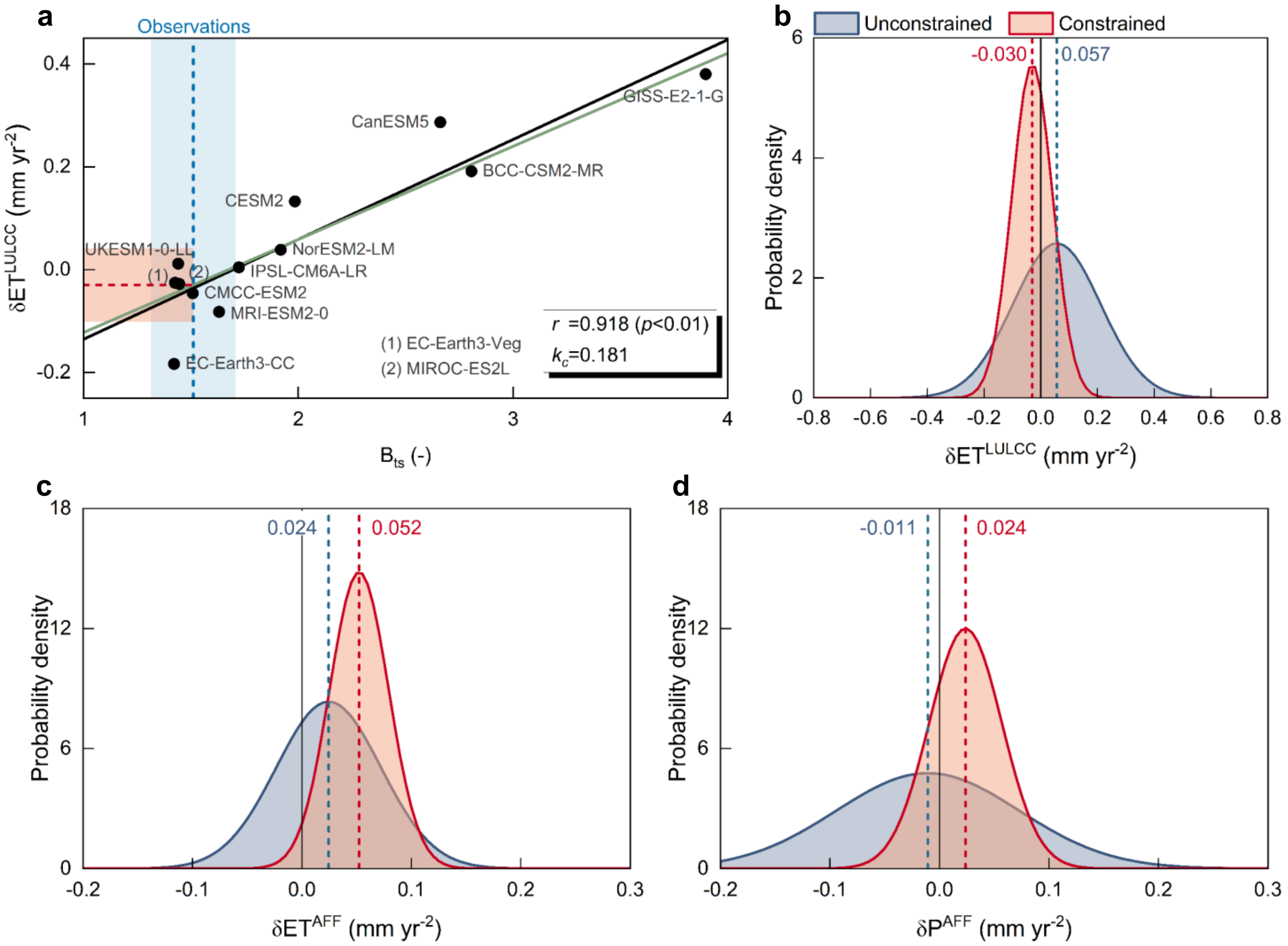

Supplementary Fig. 14. Constrained estimates derived based on transpiration-specific Bowen ratio (B_{ts}) (i.e., not applying the log-transformation). (a) Emergent relationship between the modelled effect of land use and land cover change on annual evapotranspiration (δET^{LULCC}) and the modelled global averaged transpiration-specific Bowen ratio (B_{ts}) during the period 1982-2014. LULCC over this period is generally characterized as the conversion from forests to croplands at the global extent. Each dot denotes a CMIP6 ESM result, which corresponds to δET^{LULCC} estimate shown in Fig. 1b. The black solid line indicates the best-fit regression line across ESMs, with correlation coefficient (r) provided in the panel. The green solid line indicates an observational correction based on Eq. (5) with associated slope (k_c) provided in label. The vertical blue dashed line and shaded areas represent the observation-based estimate of B_{ts} and its uncertainty (one standard deviation). Such observation-based estimate is derived from an eight-member ensemble of observation-based combined datasets (Methods). The horizonal red dashed line and shaded areas show the resulting constrained estimate of δET^{LULCC} and its uncertainty based on the hierarchical emergent constraint approach (Methods). (b) The probability density functions of global averaged δET^{LULCC} for the original results of CMIP6 ESMs (blue) and the observationally constrained results (red). (c) Same as (b), but for the global averaged effect of future (2015-2099) afforestation on annual evapotranspiration (δET^{AFF}). (d) The probability density functions of global averaged effect of future (2015-2099) afforestation on annual precipitation (δP^{AFF}) for the original results of CMIP6 ESMs (blue) and the observationally constrained results (red) by Eq. (10). Such constraint on δP^{AFF} is based on the combination of the significant linear regression shown in Supplementary Fig. 9c with (c) the constrained value of δET^{AFF}.

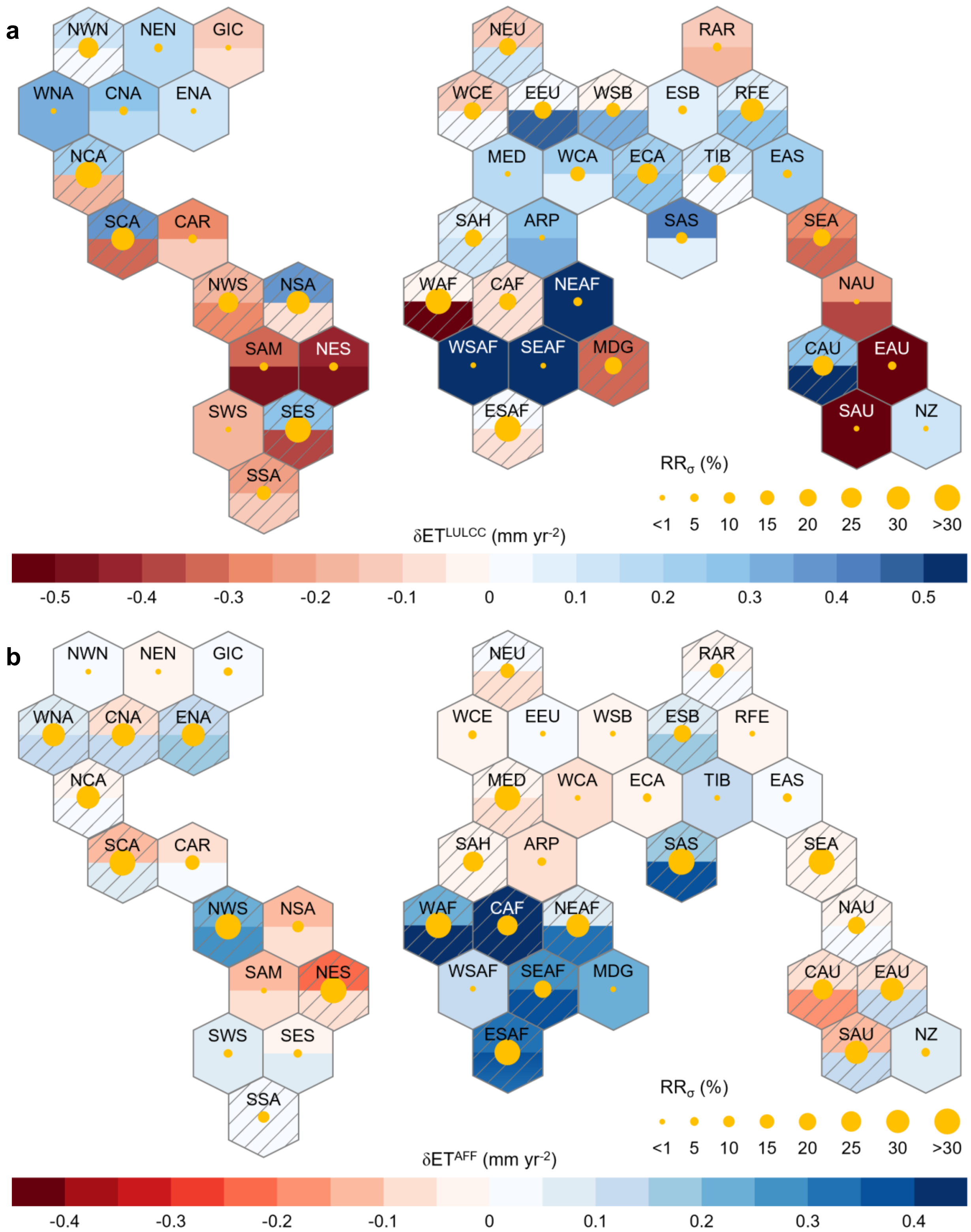

Supplementary Fig. 15. Constrained estimates over IPCC AR6 reference regions derived based on transpiration-specific Bowen ratio (B_{ts}) (i.e., not applying the log-transformation). (a) Constrained effect of land use and land cover change on terrestrial evapotranspiration (δET^{LULCC}) during 1982-2014. Within each hexagon, the filled color in the upper and lower parts represent the unconstrained estimate from the original CMIP6 model ensemble and the B_{ts} observation-constrained estimate, respectively. The size of the yellow dot indicates the relative reduction in standard deviation (RR_σ) after applying the hierarchical emergent constraint approach. The hexagons with the diagonal lines indicate that the emergent relationship is statistically significant at the 90% confidence level. Detailed information of these reference regions is provided in Supplementary Fig. 8. (b) Same as (a), but for the constrained future afforestation impact on terrestrial evapotranspiration (δET^{AFF}) during 2015-2099.

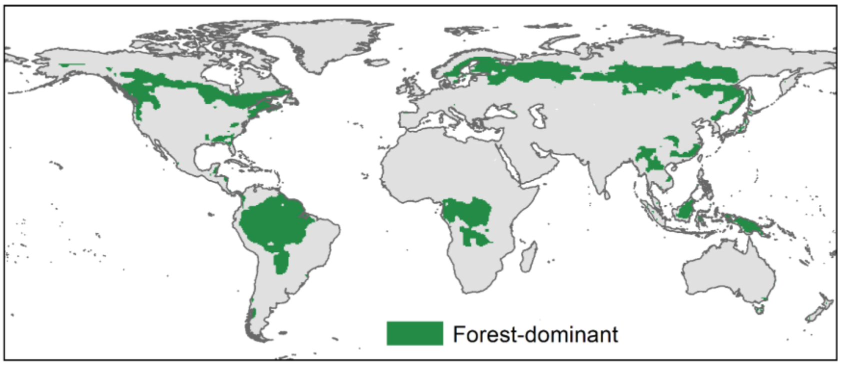

Supplementary Fig. 16. Spatial distribution of areas dominated by forests. These forest-dominant areas are identified as grid-cells with mean annual cover fraction of trees ≥ 60% during the period 1982-2014, as derived from the CMIP6 model ensemble mean. ArcGIS Pro was used as technical tool for data processing and visualizing this figure. However, no ESRI basemaps or proprietary ESRI datasets, and no screenshots of the ArcGIS software interface were used.

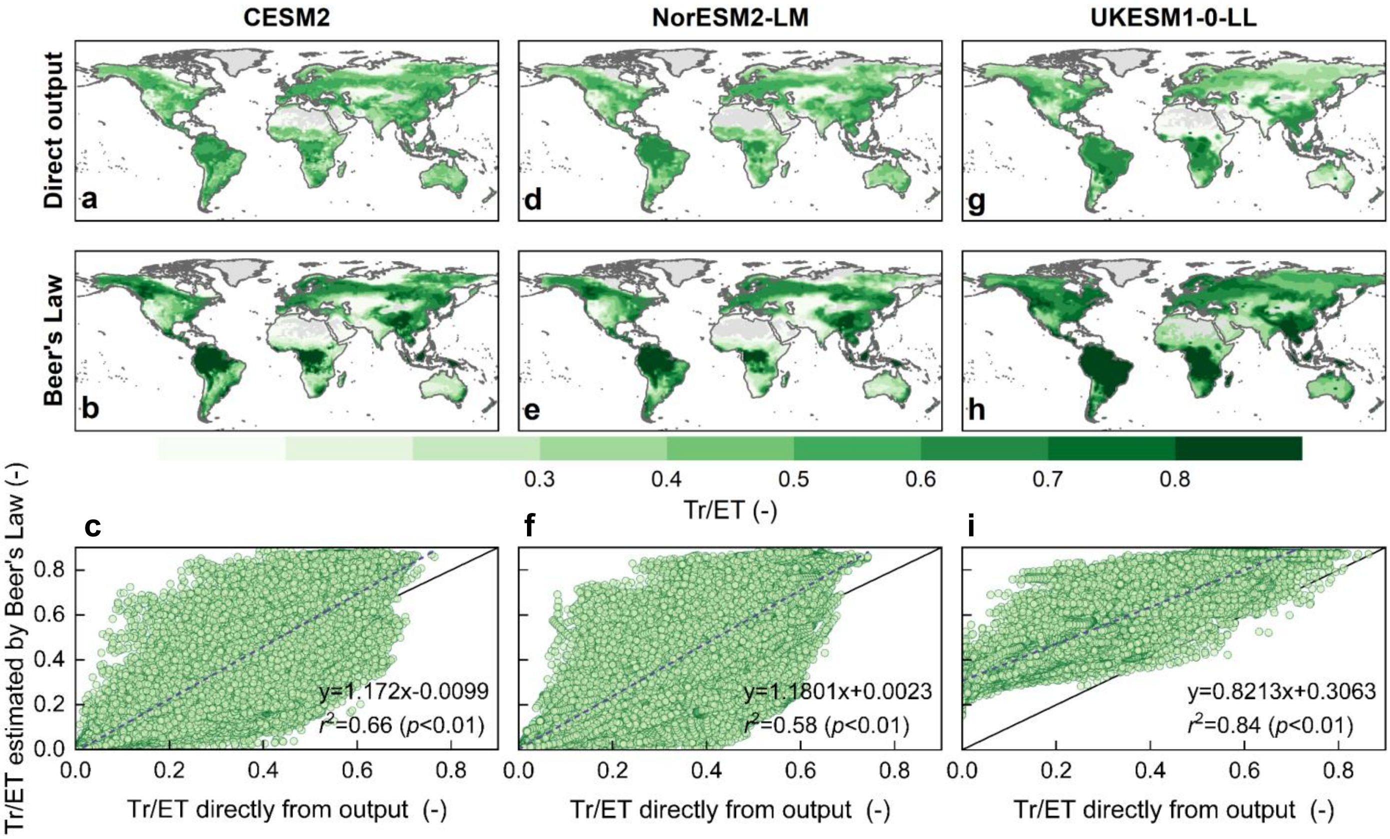

Supplementary Fig. 17. Performance evaluation of Beer's Law in estimating ratio of transpiration to terrestrial evapotranspiration (Tr/ET). (a) Spatial patterns of mean annual Tr/ET during the period 1982-2014, as directly derived from CESM2 output under the "historical" experiment. (b) Same as (b), but as estimated by Beer's Law in combination with leaf area index (LAI) modelled by CESM2 under the "historical" experiment. (c) Comparison of Tr/ET derived directly from CESM2 output against that estimated by Beer's Law. Each symbol represents one grid-cell. Purple dashed lines indicate the best-fit with equation provided in each panel. (d-f, g-i) Same as (a-c), but for NorESM2-LM and UKESM1-0-LL, respectively. ArcGIS Pro was used as technical tool for data processing and visualizing this figure. However, no ESRI basemaps or proprietary ESRI datasets, and no screenshots of the ArcGIS software interface were used.

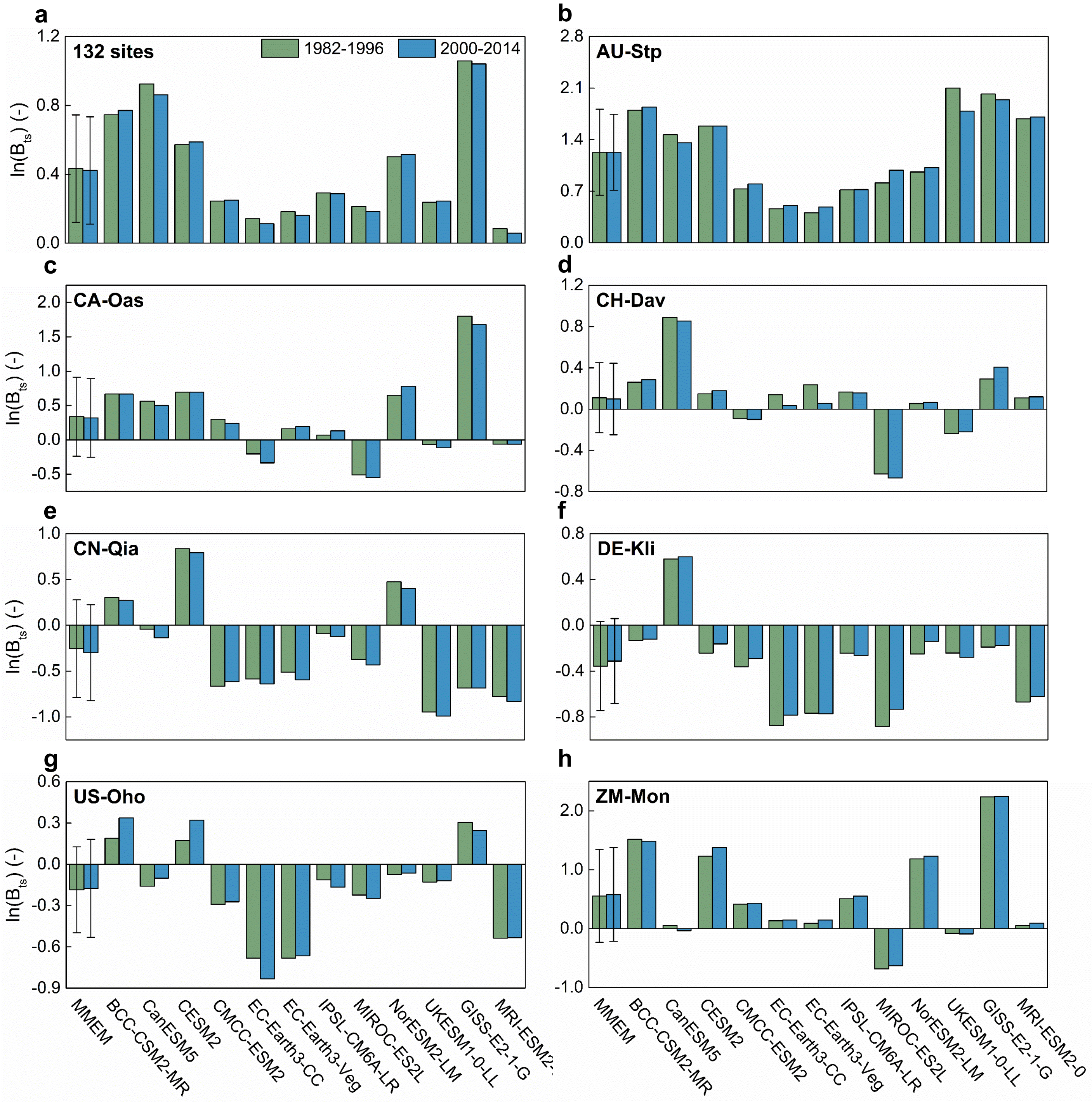

Supplementary Fig. 18. Transpiration-specific Bowen ratio (B_{ts}) for site locations during the periods 1982-1996 and 2000-2014 derived from CMIP6 ESMs. (a) Natural logarithm value of B_{ts} ($\ln(B_{ts})$) averaged over 132 FLUXNET sites, modelled by twelve individual ESMs and their ensemble mean (MMEM). Error bars represent the standard deviation of $\ln(B_{ts})$ derived from ensemble members (i.e., twelve CMIP6 ESMs). (b-h) Same as (a), but for specific sites which spread over continents. Detailed information of these sites is provided in Supplementary Data 1 and 2. Modelled B_{ts} and $\ln(B_{ts})$ for each site is extracted based on the grid-cell corresponding to the longitude and latitude of site within 0.5°×0.5° spatial resolution.

Supplementary Table 1. Information of CMIP6 ESMs used in this study.

Model name	Land surface component	Modelling Center	Default realization
BCC-CSM2-MR	BCC_AVIM	Beijing Climate Center of China Meteorological Administration, China	r1i1p1f1
CanESM5	CLASS-CTEM	Canadian Centre for Climate Modelling and Analysis, Canada	r1i1p1f1
CESM2	CLM5	National Center for Atmospheric Research, USA	r1i1p1f1, r10i1p1f1 for future [1]
CMCC-ESM2	CLM4.5	Fondazione Centro Euro-Mediterraneo sui Cambiamenti Climatici, Italy	r1i1p1f1
EC-Earth3-CC	HTESSEL with LPJ-GUESS	EC-Earth Consortium, Europe	r1i1p1f1
EC-Earth3-Veg	HTESSEL with LPJ-GUESS	EC-Earth Consortium, Europe	r1i1p1f1
GFDL-ESM4	LM4.1	NOAA Geophysical Fluid Dynamics Laboratory, USA	r1i1p1f1
GISS-E2-1-G	GISS LSM	NASA Goddard Institute for Space Studies, USA	r1i1p3f1
IPSL-CM6A-LR	ORCHIDEE	Institut Pierre-Simon Laplace, France	r1i1p1f1
MIROC-ES2L	MATSIRO with VISIT-e	Japan Agency for Marine-Earth Science and Technology, Japan	r1i1p1f2
MRI-ESM2-0	HAL1.0	Meteorological Research Institute of the Japan Meteorological Agency, Japan	r1i1p1f1
NorESM2-LM	CLM5	Norwegian Climate Center, Norway	r1i1p1f1
UKESM1-0-LL	JULES-ES-1.0	Met Office Hadley Centre, UK	r1i1p1f2

[1]: According to information from CESM2 staff, the original "r1i1p1f1" for CESM2 future projections has been retracted and further resubmitted with new name "r10i1p1f1". see: https://bb.cgd.ucar.edu/cesm/threads/query-about-cmip6-cesm2-model-under-ssp585.5718/.

Supplementary Table 2. Description of CMIP6 factorial simulations. For CMIP6, land use and land cover state for a specific year (e.g., 1850) and their changes during historical and future scenario periods are all derived from Land-Use Harmonization 2 (LUH2) dataset[16].

Simulation name	Type	Forcing constraints	
		LULCC forcing	Other forcings
historical (1850-2014)	Fully-coupled mode	Land use and land cover vary over time	Factors including CO_2, CH_4, N_2O, and aerosols vary over time
hist-noLu (1850-2014)	Fully-coupled mode	Land use and land cover fixed at 1850 state	Factors including CO_2, CH_4, N_2O, and aerosols vary over time
histSST (1850-2014)	Ocean-uncoupled mode	Land use and land cover vary over time	Factors including CO_2, CH_4, N_2O, and aerosols vary over time, but sea-surface temperature and sea ice are historically prescribed instead of simulated
histSST-noLu (1850-2014)	Ocean-uncoupled mode	Land use and land cover fixed at 1850 state	Factors including CO_2, CH_4, N_2O, and aerosols vary over time, but sea-surface temperature and sea ice are historically prescribed instead of simulated
ssp370 (2015-2099)	Fully-coupled mode	Land use and land cover vary over time, and is taken from ssp370 (deforestation scenario)	Factors including CO_2, CH_4, N_2O, and aerosols vary over time, and are taken from ssp370
ssp370-ssp126Lu (2015-2099)	Fully-coupled mode	Land use and land cover vary over time, but is taken from ssp126 (afforestation scenario)	Factors including CO_2, CH_4, N_2O, and aerosols vary over time, and are taken from ssp370

Supplementary Table 3. List of factorial experiments that each ESM participates in.

Model name	Participating experiments
BCC-CSM2-MR	historical, hist-noLu, ssp370, ssp370-ssp126Lu
CanESM5	historical, hist-noLu, ssp370, ssp370-ssp126Lu
CESM2	historical, hist-noLu, ssp370, ssp370-ssp126Lu
CMCC-ESM2	historical, hist-noLu, ssp370, ssp370-ssp126Lu
EC-Earth3-CC	historical, hist-noLu
EC-Earth3-Veg	historical, hist-noLu
GFDL-ESM4	historical, ssp370, ssp370-ssp126Lu
GISS-E2-1-G	historical, histSST, histSST-noLu
IPSL-CM6A-LR	historical, hist-noLu, ssp370, ssp370-ssp126Lu
MIROC-ES2L	historical, hist-noLu, ssp370, ssp370-ssp126Lu
MRI-ESM2-0	historical, histSST, histSST-noLu
NorESM2-LM	historical, hist-noLu, ssp370, ssp370-ssp126Lu
UKESM1-0-LL	historical, hist-noLu, ssp370, ssp370-ssp126Lu

Supplementary Table 4. Inter-model correlation between the effect of land use and land cover change on evapotranspiration (δET^{LULCC}) and the natural logarithm of transpiration-specific Bowen ratio ($\ln(B_{ts})$) at the global mean scale for the period 1982-2014, after excluding the specific model. For each sensitivity experiment by leaving specific model out of the emergent relationship, the associated corrected slope k_c by Eq. (6) within the emergent constraint framework and the resulting constrained estimate of δET^{LULCC} and the relative reduction in standard deviation (RR_σ, %) between the constrained δET^{LULCC} with the unconstrained one from original CMIP6 model ensemble by Eq. (9) are provided below. Constrained values of δET^{LULCC} are expressed as mean ± standard deviation.

Model left out	Correlation coefficient r	Corrected slope k_c	Constrained value (mm yr^{-2})	RR_σ (%)
BCC-CSM2-MR	0.932 (p<0.01)	0.405	-0.033±0.085	48.3
CanESM5	0.928 (p<0.01)	0.368	-0.037±0.079	48.2
CESM2	0.935 (p<0.01)	0.394	-0.038±0.083	50.5
CMCC-ESM2	0.928 (p<0.01)	0.394	-0.032±0.085	49.0
EC-Earth3-CC	0.946 (p<0.01)	0.365	-0.014±0.073	51.5
EC-Earth3-Veg	0.934 (p<0.01)	0.403	-0.038±0.084	49.6
IPSL-CM6A-LR	0.931 (p<0.01)	0.395	-0.032±0.085	49.8
MIROC-ES2L	0.932 (p<0.01)	0.401	-0.037±0.085	49.4
NorESM2-LM	0.933 (p<0.01)	0.396	-0.032±0.085	50.1
UKESM1-0-LL	0.944 (p<0.01)	0.410	-0.043±0.082	51.2
GISS-E2-1-G	0.884 (p<0.01)	0.361	-0.031±0.084	36.6
MRI-ESM2-0	0.938 (p<0.01)	0.387	-0.024±0.080	50.7

Supplementary Table 5. Constrained estimates of the global effect of land use and land cover change on evapotranspiration (δET^{LULCC}) during the period 1982-2014, derived from the combination of ESM-based emergent relationship (Fig. 2a) with the different subsets of observation-based datasets. To maximum the simplicity, in first row, 20CR and ERA5-Land are represented as C and E, and GLEAM v3.8a, Gerrits' model, LAI-based upscaling, and PML-V2 are represented as G1, G2, L, and P, respectively. To this end, the combination of 20CR and GLEAM v3.8a is simply shown as CG1, and so on. Constrained values are expressed as mean ± standard deviation. The associated corrected slope k_c by Eq. (6) and the relative reduction in standard deviation (RR_σ, %) between the constrained δET^{LULCC} estimated by associated subset with the unconstrained one from CMIP6 model ensemble by Eq. (9) are also provided below.

Subset	Corrected slope k_c	Constrained value (mm yr^{-2})	RR_σ (%)
CL-CP-EG1-EG2-EL-EP	0.376	-0.018±0.083	46.3
CG2-CP-EG1-EG2-EL-EP	0.386	-0.029±0.081	48.0
CG2-CL-EG1-EG2-EL-EP	0.385	-0.028±0.081	47.8
CG2-CL-CP-EG2-EL-EP	0.436	-0.012±0.065	58.1
CG2-CL-CP-EG1-EL-EP	0.379	-0.016±0.082	46.8
CG2-CL-CP-EG1-EG2-EP	0.379	-0.024±0.082	46.8
CG2-CL-CP-EG1-EG2-EL	0.379	-0.023±0.083	46.7
CG1-CP-EG1-EG2-EL-EP	0.366	-0.036±0.086	44.4
CG1-CL-EG1-EG2-EL-EP	0.364	-0.035±0.086	44.2
CG1-CL-CP-EG2-EL-EP	0.392	-0.018±0.079	49.2
CG1-CL-CP-EG1-EL-EP	0.353	-0.022±0.089	42.3
CG1-CL-CP-EG1-EG2-EP	0.358	-0.031±0.088	43.2
CG1-CL-CP-EG1-EG2-EL	0.357	-0.031±0.088	43.0
CG1-CG2-EG1-EG2-EL-EP	0.382	-0.048±0.082	47.4
CG1-CG2-CP-EG2-EL-EP	0.401	-0.029±0.076	50.9
CG1-CG2-CP-EG1-EL-EP	0.365	-0.033±0.086	44.4
CG1-CG2-CP-EG1-EG2-EP	0.375	-0.044±0.084	46.0
CG1-CG2-CP-EG1-EG2-EL	0.373	-0.043±0.084	45.7
CG1-CG2-CL-EG2-EL-EP	0.393	-0.032±0.079	49.2
CG1-CG2-CL-EG1-EL-EP	0.364	-0.032±0.087	44.2
CG1-CG2-CL-EG1-EG2-EP	0.372	-0.042±0.085	45.5
CG1-CG2-CL-EG1-EG2-EL	0.373	-0.043±0.084	45.7
CG1-CG2-CL-CP-EL-EP	0.396	-0.016±0.078	49.9
CG1-CG2-CL-CP-EG2-EP	0.395	-0.024±0.078	49.7
CG1-CG2-CL-CP-EG2-EL	0.394	-0.023±0.078	49.5
CG1-CG2-CL-CP-EG1-EP	0.358	-0.029±0.088	43.2
CG1-CG2-CL-CP-EG1-EL	0.357	-0.028±0.088	43.0
CG1-CG2-CL-CP-EG1-EG2	0.364	-0.038±0.086	44.2

Supplementary Table 6. Constrained projection of future afforestation impact on terrestrial water cycle after excluding the specific model. For each sensitivity experiment by leaving specific model out of the emergent relationship, the associated inter-model correlation (r) between the effect of future (2015-2099) on annual evapotranspiration (δET^{AFF}) and the historical (1982-2014) natural logarithm value of global averaged transpiration-specific Bowen ratio ($\ln(B_{ts})$), and that between δET^{AFF} and the effect of future (2015-2099) on annual precipitation (δP^{AFF}), and the resulting constrained estimate of δET^{AFF}, δP^{AFF}, and the ultimate effect on terrestrial water availability ($\delta WA^{AFF} = \delta P^{AFF} - \delta ET^{AFF}$, see Eq. (11)) are provided below. Constrained values of δET^{AFF} and δP^{AFF} are expressed as mean ± standard deviation.

Model left out	$r(\ln(B_{ts}), \delta ET^{AFF})$	$r(\delta ET^{AFF}, \delta P^{AFF})$	Constrained value (mm yr^{-2})		
			δET^{AFF}	δP^{AFF}	δWA^{AFF}
BCC-CSM2-MR	-0.854 (p<0.01)	0.712 (p<0.05)	0.052±0.033	0.022±0.046	-0.030
CanESM5	-0.845 (p<0.01)	0.622 (p<0.10)	0.052±0.025	0.024±0.035	-0.028
CESM2	-0.889 (p<0.01)	0.709 (p<0.05)	0.050±0.033	0.023±0.041	-0.027
CMCC-ESM2	-0.890 (p<0.01)	0.708 (p<0.05)	0.055±0.033	0.023±0.040	-0.032
GFDL-ESM4	-0.879 (p<0.01)	0.747 (p<0.05)	0.052±0.032	0.034±0.042	-0.019
IPSL-CM6A-LR	-0.887 (p<0.01)	0.788 (p<0.05)	0.054±0.033	0.039±0.039	-0.016
MIROC-ES2L	-0.861 (p<0.01)	0.707 (p<0.05)	0.047±0.032	0.024±0.043	-0.024
NorESM2-LM	-0.929 (p<0.01)	0.683 (p<0.10)	0.048±0.029	0.013±0.034	-0.034
UKESM1-0-LL	-0.866 (p<0.01)	0.723 (p<0.05)	0.051±0.033	0.005±0.032	-0.045

Supplementary Table 7. Description of observation-based gridded products used in this study. To be consistent with the historical period in CMIP6 project, time series from 1982 to 2014 of these products are extracted and used for our analyses. Access information of these products has been provided in Methods.

Variable	Product name	Spatial coverage	Temporal coverage	Spatial resolution	Temporal resolution
ET and Tr	Gerrits' model	Global	1982-2010s (presumedly) [1]	0.25º×0.25º	long-term mean annual
	GLEAM v3.8a	Global	1980-2022	0.25º×0.25º	monthly/annual
	LAI-based upscaling	Global	1982-2014	1º×1º	monthly
	PML-V2	Global	1982-2014	0.05º×0.05º	monthly
Rn	20CR v3	Global	1981-2015	1º×1º	monthly
	ERA5-Land	Global	1950-present	0.1º×0.1º	monthly
H	FLUXCOM-CRUNCEP	Global	1950-2016	0.5º×0.5º	monthly
	FLUXCOM-G3WP3	Global	1950-2014	0.5º×0.5º	monthly

[1]: Temporal coverage of estimates from Gerrits' model is not explicitly stated in associated study[17], but is presumed to roughly cover the historical period we focused on according to the time coverage of the input data reported in the article.

Supplementary Table 8. Mean annual sensible heat (H) at the global scale during the period 1982-2014. The first eight columns (from left to right) show the H (W m^{-2}) estimated by the different combinations of four observation-based evapotranspiration products and two climate reanalysis datasets (details in Methods). In the ensemble configuration (the ninth column), value is expressed as ensemble mean ± standard deviation. The last two columns show the H derived from FLUXCOM datasets. FLUXCOM-CRUNCEP and FLUXCOM-GSWP3 represent datasets derived based on CRUNCEP v8 and GSWP3 climate forcing data, respectively.

20CR				ERA5-Land				Ensemble mean	FLUXCOM (CRUNCEP)	FLUXCOM (GSWP3)
GLEAM	Gerrits' model	LAI-based upscaling	PML-V2	GLEAM	Gerrits' model	LAI-based upscaling	PML-V2			
39.24	43.63	41.70	40.48	37.33	41.72	39.79	38.56	40.31±1.88	42.13	40.83

Supplementary References

1. Pastorello, G. et al. The FLUXNET2015 dataset and the ONEFlux processing pipeline for eddy covariance data. *Sci. Data* **7**, 225 (2020).
2. Nelson, J. et al. Ecosystem transpiration and evaporation: Insights from three water flux partitioning methods across FLUXNET sites. *Glob. Change Biol.* **26**, 6916-6930 (2020).
3. Zhou, S., Yu, B., Zhang, Y., Huang, Y., & Wang, G. Partitioning evapotranspiration based on the concept of underlying water use efficiency: ET partitioning. *Water Resour. Res.* **52(2)**, 1160–1175 (2016).
4. Perez-Priego, O. et al. Partitioning eddy covariance water flux components using physiological and micrometeorological approaches. *J. Geophys. Res. Biogeosci.* **123**, 3353-3370 (2018).
5. Nelson, J. et al. Coupling water and carbon fluxes to constrain estimates of transpiration: The TEA algorithm. *J. Geophys. Res. Biogeosci.* **123**, 3617-2632 (2018).
6. Sanderson, B. et al. The potential for structural errors in emergent constraints. *Earth Syst. Dynam.* **12**, 899-918 (2021).
7. Keenan, T. et al. A constraint on historic growth in global photosynthesis due to rising CO_2. *Nat. Clim. Change* **13**, 1376-1381 (2023).
8. Lian, X. et al. Partitioning global land evapotranspiration using CMIP5 models constrained by observations. *Nat. Clim. Change* **8**, 640-646 (2018).
9. Seland, Ø. et al. Overview of the Norwegian Earth System Model (NorESM2) and key climate response of CMIP6 DECK, historical, and scenario simulations. *Geosci. Model Dev.* **13**, 6165-6200 (2020).
10. Bowman, K., Cressie, N., Qu, X. & Hall, A. hierarchical statistical framework for emergent constraints: Application to snow-albedo feedback. *Geophys. Res. Lett.* **45**, 13050-13059 (2018).
11. Cox, P. et al. Sensitivity of tropical carbon to climate change constrained by carbon dioxide variability. *Nature* **494**, 341-344 (2013).
12. Winkler, A., Myneni, R., Alexandrov, G. & Brovkin, V. Earth system models underestimate carbon fixation by plants in the high latitudes. *Nat. Commun.* **10**, 885 (2019).
13. Hoek van Dijke, A. et al. Shifts in regional water availability due to global tree restoration. *Nat. Geosci.* **15**, 363-368 (2022).
14. Meier, R. et al. Empirical estimate of forestation-induced precipitation changes in Europe. *Nat. Geosci.* **14**, 473-478 (2021).
15. Iturbide, M. et al. An update of IPCC climate reference regions for subcontinental analysis of climate model data: definition and aggregated datasets. *Earth Syst. Sci. Data* **12**, 2959-2970 (2020).
16. Hurtt, G. et al. Harmonization of global land use change and management for the period 850-2100 (LUH2) for CMIP6. *Geosci. Model Dev.* **13**, 5425-5464 (2020).
17. Mianabadi, A., Coenders-Gerrits, M., Shirazi, P., Ghahraman, B. & Alizadeh, A. A global Budyko model to partition evaporation into interception and transpiration. *Hydrol. Earth Syst. Sci.* **23**, 4983-5000 (2019).
