# Supplementary material for: Emergent constraints on the hydrological impacts of land use and land cover change
Source: Nat Commun. 2026 Feb 18;17:2908. doi: 10.1038/s41467-026-69883-2 (PMC13031313; doi:10.1038/s41467-026-69883-2)
Supplement: Supplementary file 2 — Description of Additional Supplementary Files [file 41467_2026_69883_MOESM2_ESM.pdf]

Description of Additional Supplementary Files

Supplementary Data 1. Basic information of 132 FLUXNET sites where eddy covariance measurements are collected.

Supplementary Data 2. Mean annual sensible heat (H), latent heat (LE), and transpiration (Tr) for 132 FLUXNET sites globally. H and LE, as well as their uncertainties (represented by relative standard deviation (SD)) are collected directly from FLUXNET dataset1. Tr is obtained from Nelson et al.2, which is estimated by the combination of site measurements with three different water flux partitioning methods including the underlying water use efficiency (uWUE) method, the Pérez-Priego method, and the Transpiration Estimation Algorithm (TEA) method.

Supplementary Data 3. Effects of historical (1982-2014) land use and land cover change on annual evapotranspiration (δET^{LULCC}) in the forty-four IPCC AR6 reference regions. δFA and δCA represent the trends in mean annual forest area and cropland area during 1982-2014, respectively, as derived from the CMIP6 model ensemble. RC^{LULCC}, namely the relative contribution of LULCC on historical ET change, is calculated as the ratio between mean value of unconstrained δET^{LULCC} and mean trend in ET in “historical” experiment derived from original CMIP6 model ensemble mean. Unconstrained estimates of δET^{LULCC} from the original CMIP6 model ensemble (Eqs. (1) and (2)), and constrained estimates of δET^{LULCC} derived based on the hierarchical emergent constraint approach (Eq. (5)) are shown as follows. Values of δFA, δCA and δET^{LULCC} are expressed as mean ± standard deviation. For each region, inter-model correlation between δET^{LULCC} and the natural logarithm of transpiration-specific Bowen ratio ($\ln(B_{ts})$) during the period 1982-2014, and the associated corrected slope k_c by Eq. (6) within the emergent constraint framework, as well as the relative reduction in standard deviation (RR_{σ}, %) between the constrained δET^{LULCC} with the unconstrained one by Eq. (9) are also provided below. Detailed information of these regions is provided in Supplementary Fig. 8.

Supplementary Data 4. Effects of future (2015-2099) afforestation on terrestrial water cycle in the forty-four IPCC AR6 reference regions. ΔFA represents the difference in mean annual forest area fraction during 2015-2099 between the two scenarios (SSP1-2.6 versus SSP3-7.0) derived from CMIP6 model ensemble mean. Statistical significance of the difference is assessed by *t*-test. Unconstrained values of effect of future afforestation on evapotranspiration (δET^{AFF}) are computed by Eq. (3). Similar method is also used to derive the unconstrained value of effect of future afforestation on precipitation (δP^{AFF}). RC^{AFF}, namely the relative contribution of afforestation on future ET change, is calculated as the ratio between mean value of unconstrained δET^{AFF} and mean trend in ET in “ssp370-ssp126Lu” experiment derived from original CMIP6 model ensemble mean. Correspondingly, constrained values of δET^{AFF} and δP^{AFF} derived based on the hierarchical emergent constraint approach (Eqs. (5) and (10)) are also provided at the regional scale. Values of δET^{AFF} and δP^{AFF} are expressed as mean ± standard deviation. Unconstrained (constrained) value of effect on terrestrial water availability (δWA^{AFF}) is further estimated as the difference between unconstrained (constrained) δP^{AFF} and δET^{AFF}. Detailed information of these regions is provided in Supplementary Fig. 8. In fact, the so-called “afforestation” is not applicable to the small parts of the globe including Greenland/Iceland (GIC), Eastern Europe (EEU), Mediterranean (MED), Russian Far East (RFE) and East Asia (EAS), where forest cover in SSP1-2.6 is overall lower than that in SSP3-7.0. Nevertheless, given the generality of afforestation across the globe, we consistently refer to δET^{AFF} as the trend in ET induced by future afforestation.

Supplementary Data 5. Description of the hierarchical emergent constraint framework applied for the forty-four IPCC AR6 reference regions. For each region, inter-model correlation (*r*) between the effect of future (2015-2099) on annual evapotranspiration (δET^{AFF}) and the historical (1982-2014) natural logarithm value of global averaged transpiration-specific Bowen ratio ($\ln(B_{ts})$), and that between δET^{AFF} and the effect of future (2015-2099) on annual precipitation (δP^{AFF}) are provided below. Meanwhile, the associated corrected slope k_c by Eq. (6) and the relative reduction in standard deviation (RR_σ, %) between the constrained δET^{AFF} with the originally unconstrained one by Eq. (9) are derived at the regional scale and also shown below. Detailed information of these regions is provided in Supplementary Fig. 8.
